# Synthesis of LaCN_3_, TbCN_3_, CeCN_5_, and TbCN_5_ Polycarbonitrides at Megabar Pressures

**DOI:** 10.1021/jacs.4c06068

**Published:** 2024-06-25

**Authors:** Andrey Aslandukov, Akun Liang, Amanda Ehn, Florian Trybel, Yuqing Yin, Alena Aslandukova, Fariia I. Akbar, Umbertoluca Ranieri, James Spender, Ross T. Howie, Eleanor Lawrence Bright, Jonathan Wright, Michael Hanfland, Gaston Garbarino, Mohamed Mezouar, Timofey Fedotenko, Igor A. Abrikosov, Natalia Dubrovinskaia, Leonid Dubrovinsky, Dominique Laniel

**Affiliations:** † Bavarian Research Institute of Experimental Geochemistry and Geophysics (BGI), University of Bayreuth, 95440 Bayreuth, Germany; ‡ Material Physics and Technology at Extreme Conditions, Laboratory of Crystallography, University of Bayreuth, 95440 Bayreuth, Germany; § Centre for Science at Extreme Conditions and School of Physics and Astronomy, University of Edinburgh, EH9 3FD Edinburgh, United Kingdom; ∥ Department of Physics, Chemistry and Biology (IFM), Linköping University, SE-581 83 Linköping, Sweden; ⊥ European Synchrotron Radiation Facility, 38000 Grenoble, France; # Photon Science, Deutsches Elektronen-Synchrotron, 22607 Hamburg, Germany

## Abstract

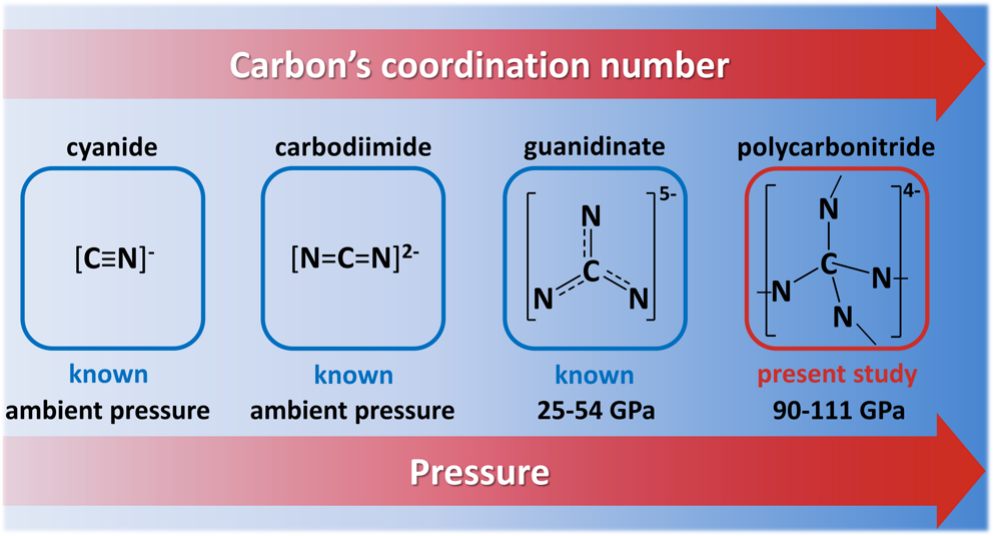

Inorganic ternary metal–C–N compounds with covalently bonded C–N anions encompass important classes of solids such as cyanides and carbodiimides, well known at ambient conditions and composed of [CN]^−^ and [CN_2_]^2–^ anions, as well as the high-pressure formed guanidinates featuring [CN_3_]^5–^ anion. At still higher pressures, carbon is expected to be 4-fold coordinated by nitrogen atoms, but hitherto, such CN_4_-built anions are missing. In this study, four polycarbonitride compounds (LaCN_3_, TbCN_3_, CeCN_5_, and TbCN_5_) are synthesized in laser-heated diamond anvil cells at pressures between 90 and 111 GPa. Synchrotron single-crystal X-ray diffraction (SCXRD) reveals that their crystal structures are built of a previously unobserved anionic single-bonded carbon–nitrogen three-dimensional (3D) framework consisting of CN_4_ tetrahedra connected via di- or oligo-nitrogen linkers. A crystal-chemical analysis demonstrates that these polycarbonitride compounds have similarities to lanthanide silicon phosphides. Decompression experiments reveal the existence of LaCN_3_ and CeCN_5_ compounds over a very large pressure range. Density functional theory (DFT) supports these discoveries and provides further insight into the stability and physical properties of the synthesized compounds.

## Introduction

In recent years, advancements in the high-pressure chemistry of nitrides and carbides have unveiled a wealth of surprises. Indeed, at pressures above 50 GPa, many exotic oligo- and polynitrides (i.e. containing N_4_^4–^, N_6_^6–^, N_8_^6–^ anions,^1,2^ N_6_ rings,^3−5^ N_18_ macrocycle,^6^ infinite one-dimensional (1D)-polynitrogen chains,^1,7−12^ and two-dimensional (2D)-polynitrogen layers^2,12^) as well as polycarbides (i.e. containing graphene-, polyacene-, and para-poly(indenoindene)-like ribbons^13−16^) were discovered. Recently discovered binary carbon nitrides C_3_N_4_ and CN_2_, built of CN_4_ tetrahedra and synthesized at pressures above 70 GPa,^17^ possess many remarkable physical properties, such as superhardness, ultraincompressibility, photoluminescence, piezoelectricity, and superconductivity, making these multifunctional materials candidates for technological applications, considering that they are recoverable to ambient conditions. A recent high-pressure study of the H–C–N system shows that the addition of another element, namely, hydrogen, helps to produce CN_4_ tetrahedra frameworks at lower pressures.^18^ The addition of metal atoms to carbon nitrides is expected to enable tuning and controlling of the physical properties of such materials. Therefore, exploration of the ternary metal–C–N systems at high pressures is very promising.

Ternary metal–C–N compounds with covalently bonded C–N anions at ambient conditions encompass important classes of solids such as cyanides (with CN^–^ anions)^19,20^ and carbodiimides (with NCN^2–^ anions).^21−27^ Recently, this family of species was extended with the discovery of the CN_3_^5–^ anion, synthesized at mild pressures (25–54 GPa) and recoverable to ambient conditions.^28,29^ Knowing that C–N compounds built of CN_4_ tetrahedra were obtained at pressures above 70 GPa,^17^ as well as the fact that mild pressures (up to 54 GPa) are insufficient for the formation of C–N anions with a tetra-coordinated carbon,^29^ the stabilization of CN_4_^8–^ units,^30^ or/and the formation of polycarbonitrides built of corner/edge-sharing CN_4_ tetrahedra in ternary metal–C–N systems are expected at pressures above 70 GPa. The discovery of such compounds might be interesting not only for chemical and materials sciences but also for planetary sciences since these solids can potentially be formed at the boundaries between the rock core and the methane-ammonia-rich mantle of ice-giant planets.

Here we present the high-pressure, high-temperature synthesis and characterization of four hitherto unknown lanthanide polycarbonitrides, LaCN_3_, TbCN_3_, CeCN_5_, and TbCN_5_, at megabar pressures. Their crystal structures were solved and refined based on synchrotron single-crystal X-ray diffraction, unveiling previously unobserved anionic single-bonded carbon–nitrogen three-dimensional (3D) frameworks consisting of CN_4_ tetrahedra connected via di- or oligo-nitrogen linkers. A crystal-chemical analysis demonstrates that polycarbonitride compounds have similarities with lanthanide silicon phosphides. Density functional theory (DFT) calculations cross-validate these discoveries and provide further insight into the stability and physical properties of these compounds.

## Results and Discussion

In this study, diamond anvil cells (DACs) were loaded with rare earth metal pieces (La, Tb, and Ce) embedded in molecular nitrogen (N_2_) or cyanuric triazide (C_3_N_12_; see the Methods Section for details). The samples were compressed to target pressures between 90 and 111 GPa, and the samples were laser-heated to temperatures reaching 2500 K (Table S1). The resulting multigrain samples, consisting of hundreds of submicron-sized crystallites of the reaction products, were studied by synchrotron single-crystal X-ray diffraction (SCXRD) at the P02.2 beamline of DESY and the ID11, ID15b, and ID27 beamlines of the ESRF. The analysis of the SCXRD data revealed the formation of four metal polycarbonitrides, LaCN_3_, TbCN_3_, CeCN_5_, and TbCN_5_. The refinement against SCXRD data (Figures S1−S4) yields very good agreement factors (see Tables S2−S5 and CIFs for the full crystallographic data). The carbon atoms in the discovered polycarbonitrides originate from the diamond anvils, well known to be able to act as a carbon source and participate in chemical reactions.^17,28,29,31−33^ The description of other reaction products, e.g. binary polynitrides, will be discussed elsewhere.

**Figure 1:**
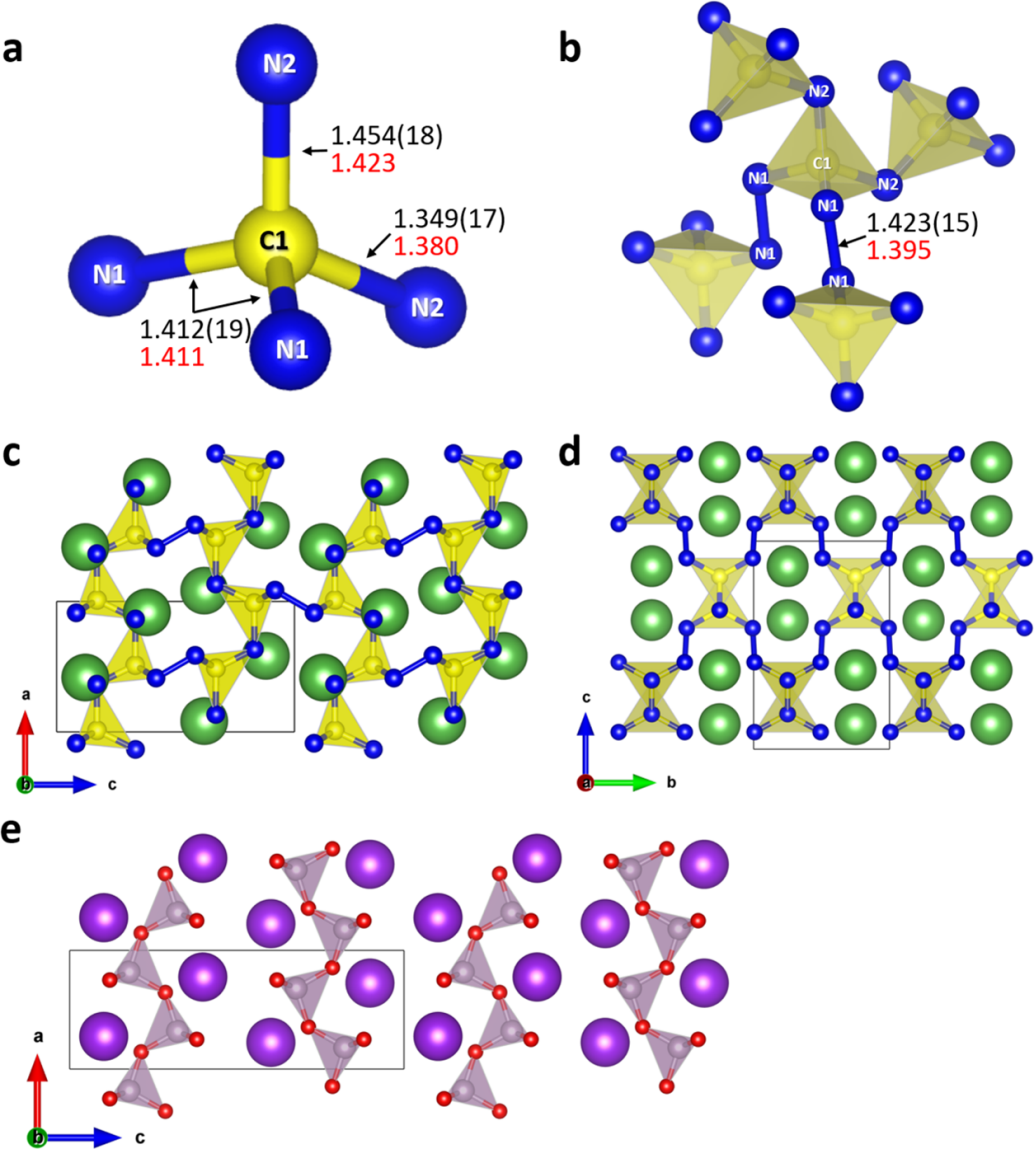
Crystal structure of LaCN_3_ at 102(2) GPa and that of the high-temperature phase of *Pnma*-KPO_3_ at ambient pressure. LaCN_3_: (a) Representation of the main building block—the CN_4_ tetrahedron. (b) Connectivity of the CN_4_ tetrahedra. Values of bond lengths obtained from the experiment are shown in black, while those obtained from the DFT calculations are shown in red. (c, d) Views along the crystallographic *b*- and *a*-axes. La atoms are shown as green spheres, C atoms as yellow spheres, and all N atoms (both N1 and N2) as blue spheres; the thin black lines outline the unit cell. (e) Crystal structure of *Pnma*-KPO_3_ at 1 atm^34^ viewed along the crystallographic *b*-axis. K atoms are shown as purple spheres, P atoms as gray spheres, and O atoms as red spheres; the thin black lines outline the unit cell.

The isostructural LaCN_3_ and TbCN_3_ solids crystallize in orthorhombic space group *Pnma* (#62). The lattice parameters of LaCN_3_ are *a* = 4.1059(12) Å, *b* = 4.870(5) Å, and *c* = 7.4758(15) Å at 102(2) GPa, while those of TbCN_3_ are *a* = 3.9813(15) Å, *b* = 4.7305(12) Å, and *c* = 7.2358(15) Å at 111(2) GPa. The crystal structure of LaCN_3_ at 102(2) GPa is described below as an example (Figure 1). LaCN_3_ consists of four crystallographically distinct atoms: La1, C1, N1, and N2 (see Table S2 and the CIF for the full crystallographic data). Carbon atoms are 4-fold coordinated by nitrogen atoms, forming irregular CN_4_ tetrahedra named mirrored sphenoids (Figure 1a). Both N1 and N2 atoms form two single covalent bonds: the N1 atoms form one N1–C1 and one N1–N1 bond between two N1 atoms of different tetrahedra, while the N2 atoms are the common nodes of corner-sharing CN_4_ tetrahedra, thus being bonded with two carbon atoms (Figure 1b). The average C–N distance within the CN_4_ tetrahedra is *d*_C–N_ = 1.407(9) Å, and the N1–N1 bond length between N1 atoms of different tetrahedra is *d*_N–N_ = 1.423(15) Å at 102(2) GPa. The average N–C–N and C–N–C/N bond angles are 109.5(4) and 111.6(10)°, respectively. Thus, all nonmetal atoms are sp^3^-hybridized. Lanthanum is 11-fold coordinated by nitrogen atoms. Since all N atoms form two single covalent bonds, each nitrogen atom has a charge of −1 in the fully ionic approximation, in turn implying the expected +3 oxidation state for the La atoms. Carbon, which makes four covalent bonds, is neutral. The bond order analysis for TbCN_3_ gives the same charge distribution as for LaCN_3_, suggesting a +3 oxidation state for Tb, one of two well-known oxidation states for this element. The repeating CN_3_^3–^ unit in LaCN_3_ and TbCN_3_, as well as its Lewis formula, are presented in Figure S5a,b.

The structure can be described as being built of corner-sharing CN_4_ tetrahedra chains running along the crystallographic *a*-axis. These chains are interconnected through the N1–N1 single bonds (Figure 1c). Each chain is linked to four other chains, producing a 3D framework (Figure 1d). Lanthanum atoms are located in the voids of this covalent 3D framework and each surrounded by nine CN_4_ tetrahedra. The structure of LaCN_3_ has similarities with that of the high-temperature *Pnma*-KPO_3_ phase.^34^ In the latter, atoms are sitting on the same Wyckoff positions as in LaCN_3_, with the nonmetal atoms forming PO_4_ tetrahedra arranged in infinite chains along the crystallographic *a-*axis through corner-sharing (Figure 1e). However, there are no covalent O–O bonds between the chains, and the distance between these PO_4_ corner-sharing chains is larger.

**Figure 2:**
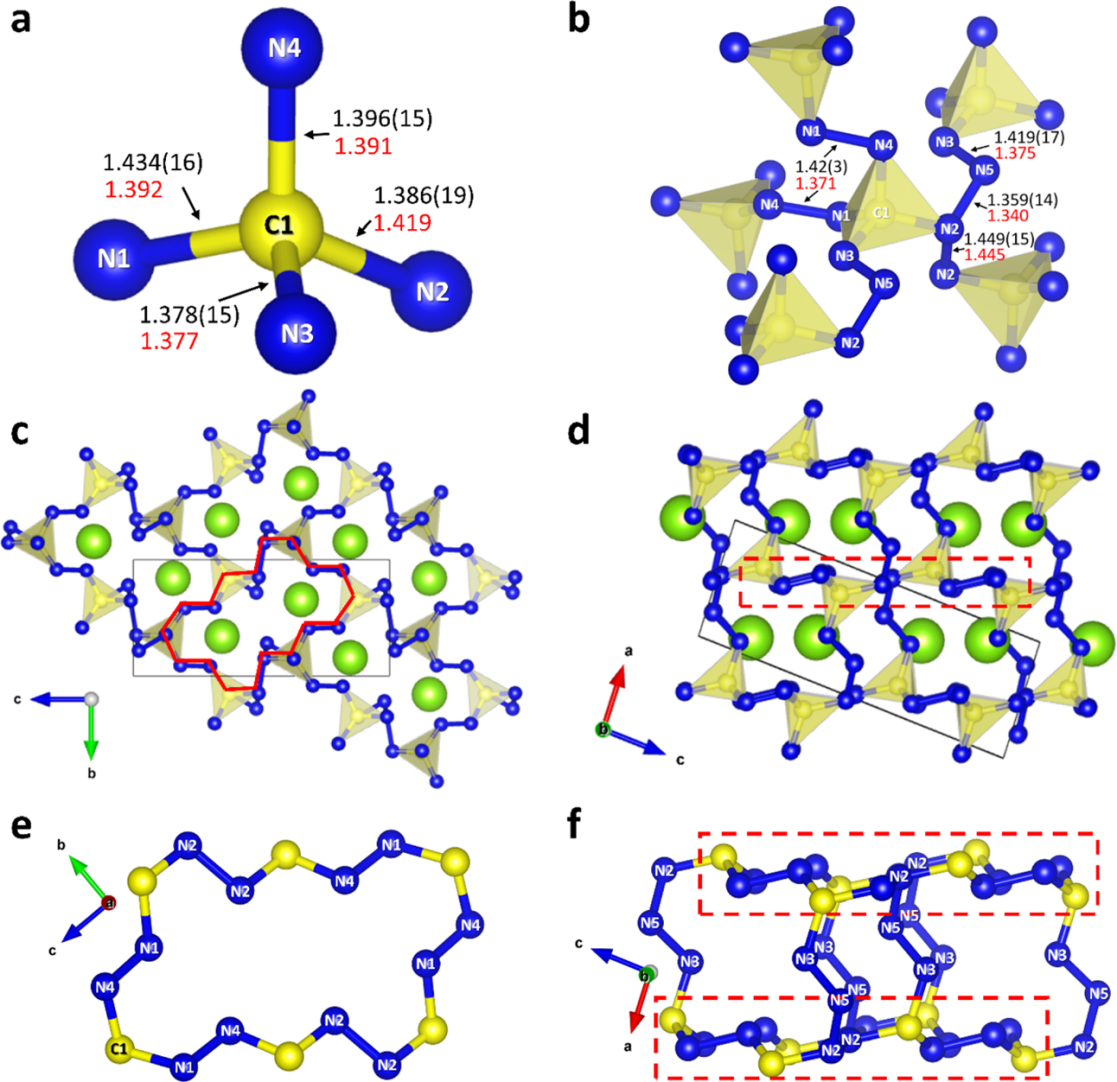
Crystal structure of CeCN_5_ at 90(2) GPa. (a) Representation of the main building block—the CN_4_ tetrahedron. (b) Connectivity of the CN_4_ tetrahedra. Values of bond lengths obtained from the experiment are shown in black, while those obtained from the DFT calculations are shown in red. (c, d) Crystal structure of CeCN_5_ viewed along the crystallographic *a*- and *b*-axes. The full red line in (c) helps visualize the atoms constituting the C_6_N_12_ rings, highlighted in (d) by a red dashed line. The thin black lines outline the unit cell. (e) Depiction of the carbonitride network’s C_6_N_12_ ring, when viewed along the crystallographic *a*-axis. (f) Two adjacent C_6_N_12_ rings interconnected through N5–N3 dimers, viewed along the crystallographic *b*-axis. Cerium, carbon, and nitrogen atoms are represented as light green, yellow, and blue spheres, respectively.

The isostructural compounds CeCN_5_ and TbCN_5_ crystallize in the monoclinic space group *P*2_1_/*n* (#14) with one lanthanide atom (Ce1 or Tb1), one carbon atom (C1), and five nitrogen atoms (N1 through N5) being crystallographically unique and all located on the 4*e* Wyckoff position (Tables S4 and S5). At a pressure of 90(2) GPa, the lattice parameters of CeCN_5_ are as follows: *a* = 3.889(1) Å, *b* = 4.740(1) Å, *c* = 10.487(6) Å, and β = 94.41(4)° (*V* = 192.72(13) Å^3^). At a pressure of 111(2) GPa, TbCN_5_ has lattice parameters of *a* = 3.8334(2) Å, *b* = 4.5221(9)Å, *c* = 10.3516(5) Å, and β = 94.699(5)° (*V* = 178.84(4) Å^3^). The crystal structure of CeCN_5_ at 90(2) GPa is described hereafter as an example of this structure type (Figure 2).

Each C1 atom in the structure of CeCN_5_ (Figure 2) is surrounded by four nitrogen atoms (N1–N4), forming a CN_4_ tetrahedron with C–N single bonds of lengths ranging from 1.378(15) to 1.434(16) Å (Figure 2a). The N–C–N bond angle varies between 104.4(10) and 119.4(13)°, with an average value of 109.5(5)°. Among the five crystallographically distinct nitrogen atoms, only N2 forms three bonds (Figure 2b): two with nitrogen atoms and one with the C1 carbon atom, for an average bond angle of 108.1(6)°. All other nitrogen atoms form two single bonds (Figure 2b): N5—with two nitrogen atoms (bond angle: 103.3(9)°), while each of the remaining three nitrogen atoms (N1, N3, and N4)—with one nitrogen atom and one carbon atom (bond angles: 109.9(10), 115.1(10), and 103.8(10)°, respectively). The 3D polycarbonitride framework in CeCN_5_ is built of CN_4_ tetrahedra linked by single covalent N2–N2 and N4–N1 bonds as well as through N2–N5–N3 trimer bridges (Figure 2b). Analogously to the LaCN_3_ and TbCN_3_ compounds, all nonmetal atoms in CeCN_5_ and TbCN_5_ are sp^3^-hybridized.

When viewed along the *a*-axis, the structure of CeCN_5_ is seen to be composed of large corrugated C_6_N_12_ rings. The rings are elongated in a direction close to [011] and arranged in a herringbone pattern (Figure 2c). Each of these C_6_N_12_ rings is constituted of six carbon atoms linked through N–N dimers (Figure 2e). There are two distinct dimers within the C_6_N_12_ rings, the N2–N2 and the N4–N1 dimers, each with a bond length of 1.449(15) and 1.422(30) Å, respectively, suggesting a single-bond character. Single-bonded N3–N5 dimers (1.419(17) Å) connect the adjacent C_6_N_12_ units so that the N3 and N5 atoms are bonded to the C1 and N2 atoms of the C_6_N_12_ rings, respectively (Figure 2d,f). Although the N2–N5 bond (1.359(14) Å) is distinctively shorter than other N–N bonds in CeCN_5_, its length is still within the single-bond lengths range.^2,35,36^ Cerium atoms, located between the layers built of C_6_N_12_ rings, are 12-fold coordinated with nitrogen, featuring an average bond length of 2.351(11) Å.

With four of the five nitrogen atoms only making two single bonds, it stands to reason that in the ionic approximation, the repeating CN_5_ unit bears a charge of −4, indicative of a +4 oxidation state for Ce, one of its two commonly observed oxidation states. In TbCN_5_, the bond order as well as the hybridization of the C and N is identical to that described in CeCN_5_. Thus, TbCN_5_ is composed of the same [CN_5_]_∞_^4–^ anion, suggesting a +4 oxidation state for Tb, which is also well known for this element. The repeating CN_5_^4–^ unit in CeCN_5_ and TbCN_5_ and its Lewis formula are presented in Figure S5c,d.

Similarly to recently discovered high-pressure C_3_N_4_ polymorphs,^17^ the common building block in LaCN_3_, TbCN_3_, CeCN_5_, and TbCN_5_ is the CN_4_ tetrahedron. The C–N single-bond lengths and N–C–N bond angles in LaCN_3_, TbCN_3_, CeCN_5_, and TbCN_5_ are also similar to those found in the C_3_N_4_ polymorphs at similar pressures. While the condensed 3D framework in the C_3_N_4_ solids is built exclusively of corner-sharing tetrahedra, the connectivity of these tetrahedra in the polynitrides discovered here is different. The 3D anionic polycarbonitride framework of LaCN_3_ and TbCN_3_ is built by corner-sharing CN_4_ as well as by N–N bonds (Figure 1b). In the CeCN_5_ and TbCN_5_ structures, the CN_4_ tetrahedra do not have common nodes and are linked only by single covalent nitrogen bonds having two types of linkers: dimers and trimers (Figure 2b).

Among the metal–C-N ternary compounds, hitherto exclusively featuring isolated anions (i.e., cyanides, carbodiimides, and guanidinates), the LaCN_3_, TbCN_3_, CeCN_5_, and TbCN_5_ solids form a novel unique class of compounds composed of a 3D single-bonded polycarbonitride framework. The geometry of the C–N anions illustrates well how high pressure stabilizes higher coordination numbers of carbon. In the ^–^N=C=N^–^ anion, which is stable under ambient conditions, the C atom is coordinated by two N atoms. At mild pressures (25–54 GPa), the CN_3_^5–^ anion is formed with carbon having a coordination number of three. Here, at higher pressures (90–111 GPa), we observe 4-fold coordinated carbon atoms in the crystal structures of LaCN_3_, TbCN_3_, CeCN_5_, and TbCN_5_. Strikingly, despite the theoretical predictions of the high-pressure stabilized CN_4_^8–^ orthonitridocarbonate anion,^30^ we do not observe its formation. This suggests that while CN_4_ units are indeed the preferred 3D building blocks, their concatenation is more favorable than preserving significantly negatively charged anions, such as CN_4_^8–^.

According to the ninth high-pressure chemistry rule of thumb formulated in 1998: *“Elements behave at high pressures like the elements below them in the periodic table at lower pressures”*,^37^ carbon and nitrogen under high pressure should behave like silicon and phosphorus at lower pressures. Therefore, one can expect similarities between the high-pressure LaCN_3_, TbCN_3_, CeCN_5_, and TbCN_5_ compounds and the ternary Ln–C–P, Ln–Si–N, and Ln–Si–P solids (Ln represents lanthanide elements) known under ambient conditions.

To date, there are no known Ln–C–P compounds with covalent C–P frameworks. As for the ternary Ln–Si–N compounds, these belong to the class of nitridosilicates,^38^ which significantly grew during the last two decades. According to the ICSD, there are six known structure types of lanthanide nitridosilicates: Eu_2_SiN_3_,^39^ Eu_2_Si_5_N_8_,^40^ Ln_5_Si_3_N_9_ (Ln = La, Ce, Pr),^41,42^ Ln_7_Si_6_N_15_ (Ln = La, Ce, Pr),^43^ Ln_3_Si_6_N_11_ (Ln = La, Ce, Pr, Nd, Sm),^44^ and LnSi_3_N_5_ (Ln = La, Ce, Nd).^44^ The crystal structures of all mentioned compounds are built of corner-sharing SiN_4_ tetrahedra forming 1D chains (Eu_2_SiN_3_), 2D layers (Ln_5_Si_3_N_9_), or 3D frameworks (Eu_2_Si_5_N_8_, Ln_7_Si_6_N_15_, Ln_3_Si_6_N_11_, and LnSi_3_N_5_). Although the building block—SiN_4_ tetrahedra—is similar to the CN_4_ building block in LaCN_3_, TbCN_3_, CeCN_5_, and TbCN_5_, the important difference is the presence of N–N linkers in the latter, which have never been observed in nitridosilicates.

**Figure 3:**
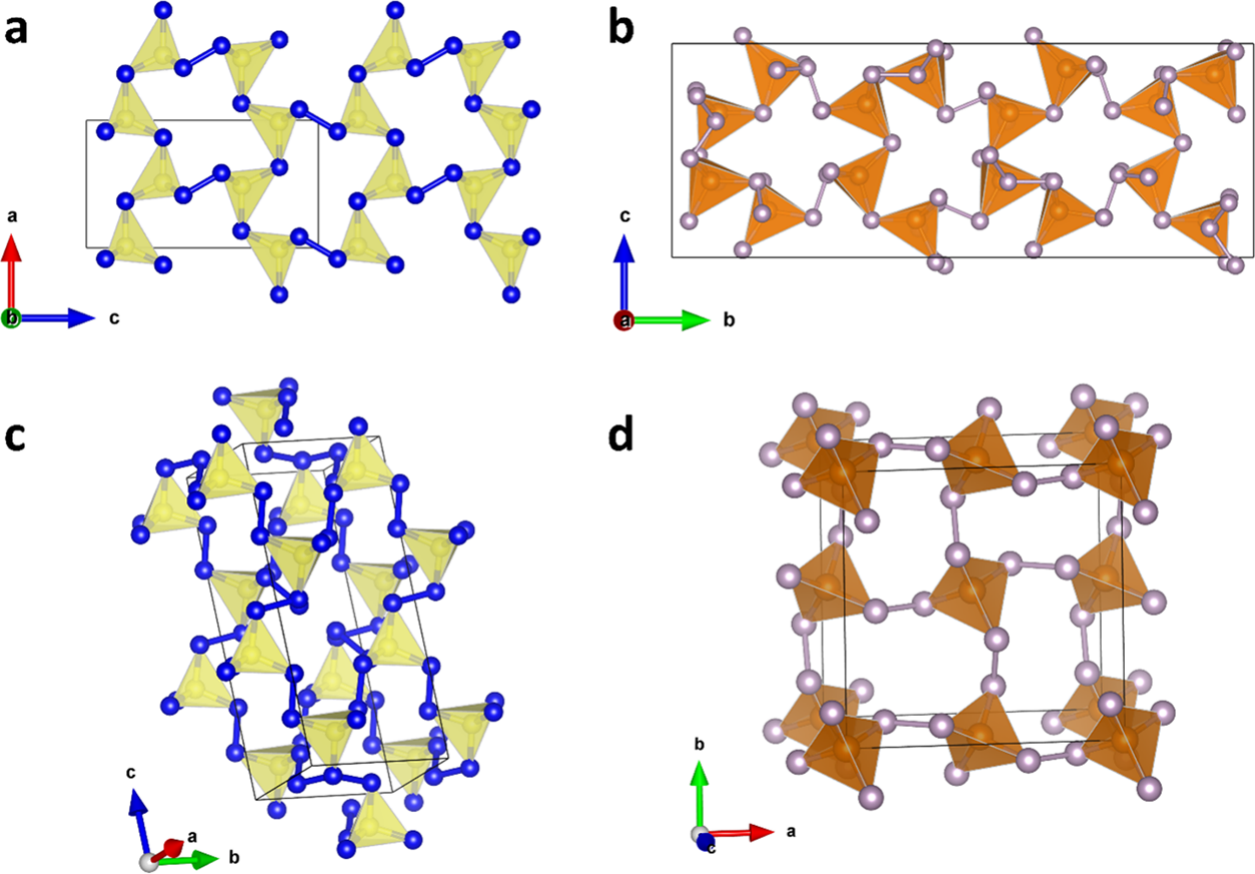
Anionic frameworks of the pairs of compounds with similar connectivity of tetrahedra: (a) LaCN_3_/TbCN_3_ and (b) *Cmc*2_1_-LaSi_2_P_6_, both built of the 3D framework of corner-sharing CN_4_ or SiP_4_ tetrahedra connected via N–N or P–P linkers; (c) CeCN_5_/TbCN_5_ and (d) *I*-42*d*-Eu_2_SiP_4_ consisting of CN_4_ or SiP_4_ tetrahedra linked exclusively via N–N or P–P bonds, producing a 3D framework. The C, N, Si, and P atoms are shown as yellow, blue, light brown, and gray balls, respectively. The thin black lines outline the unit cell.

Considering the building blocks and their connectivity, the closest analogues to the polycarbonitrides discovered here would be ternary Ln–Si–P compounds. According to the ICSD, there are four known structure types of ternary Ln–Si-P compounds built of chains, layers, or 3D frameworks of SiP_4_ tetrahedra linked, in particular, by P–P bonds: *P*2_1_/*c*-LaSiP_3_,^45^*Cmc*2_1_-LnSi_2_P_6_ (Ln = La, Ce, Pr),^46,47^*P*2_1_/*c*-La_2_SiP_4_,^48^ and *I*-42*d*-Eu_2_SiP_4_.^49^ Although none of them are isotypic to LaCN_3_/TbCN_3_ or CeCN_5_/TbCN_5_, some of the anionic motifs of these Ln–Si-P compounds have a tetrahedra connectivity similar to that observed in LaCN_3_/TbCN_3_ and CeCN_5_/TbCN_5_. Topologically, the closest analogue to LaCN_3_ and TbCN_3_ is *Cmc*2_1_-LaSi_2_P_6_,^47^ which is built of a 3D framework of corner-sharing SiP_4_ tetrahedra and P–P linkers (Figure 3a,b). The closest topological analogue to CeCN_5_ and TbCN_5_ is *I*-42*d*-Eu_2_SiP_4_,^49^ which consists of SiP_4_ tetrahedra linked exclusively via P–P bonds into a 3D framework (Figure 3c,d).

To get a deeper insight into the stability and physical properties of the novel compounds, DFT calculations were performed using the Vienna Ab initio Simulation Package (VASP)^50^ for LaCN_3_ and CeCN_5_. Due to the presence of 4f electrons in Ce and Tb, their compounds require higher-level theory. CeCN_5_ can be sufficiently well described with the DFT + *U* method.^51^ However, it has been shown that Tb is not sufficiently well described by DFT + *U*(52) and requires higher-level theory, e.g., DFT + dynamical mean field theory (DMFT).^53^ The latter is computationally very expensive and requires particular expertise in using it. For this reason, TbCN_3_ and TbCN_5_ are not investigated through theoretical calculations in this study. The relaxed structure parameters obtained from the variable-cell structure relaxations of LaCN_3_ and CeCN_5_ compounds closely reproduce the corresponding experimental values (Tables S6 and S7), confirming the validity of our computational methodology.

**Figure 4:**
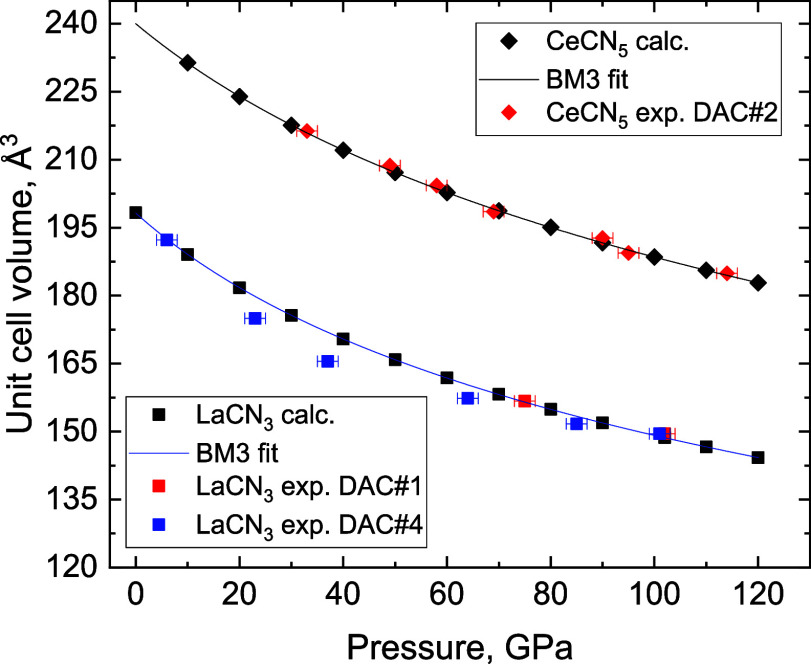
Pressure dependence of the unit cell volume of LaCN_3_ and CeCN_5_. The black symbols represent calculated data points obtained from DFT, and the red and blue symbols represent experimental data points obtained from SCXRD data. The black and blue lines are fits of the DFT data with a third-order Birch–Murnaghan equation of state. The parameters of the fit for LaCN_3_ are *V*_0_ = 198.19(4) Å^3^, *K*_0_ = 192.5(6) GPa, and *K*_p_ = 4.214(12), while for CeCN_5_, they are *V*_0_ = 239.98(9) Å^3^, *K*_0_ = 250.7(14) GPa, and *K*_p_ = 4.14(3).

To trace the structures’ behavior at lower pressures and to obtain their equation of state, full variable-cell structure relaxations for the LaCN_3_ and CeCN_5_ compounds were performed with 10 GPa pressure steps between 0 and 120 GPa (Figure 4). The lattice parameters of LaCN_3_ change monotonously in the whole pressure range (Figure S8) and the crystal structure does not undergo any significant changes, indicating the possible recoverability of LaCN_3_ to ambient conditions. That is also suggested from phonon calculations: LaCN_3_ is dynamically stable both, at the synthesis pressure and at ambient pressure (Figure S6). On the other hand, CeCN_5_ is relaxable without structural changes only down to 10 GPa, with its lattice parameters changing monotonously down to that pressure (Figure S9). CeCN_5_ is also found to be dynamically stable at its synthesis pressure of 90 and 10 GPa (Figure S7). However, below 10 GPa, full variable-cell structure relaxation resulted in a possible electronic transition in which the 4*f* electron state is promoted from above the Fermi energy to below (Figure SD1 in Supporting Discussion). It is reminiscent of the promotional model of Zachariasen and Pauling of a γ ↔ α transition in pure Ce,^54−57^ though in CeCN_5_ the transition occurs upon decompression and leads to a significant modification of the crystal structure of the compound (Figure SD2 in Supporting Discussion). The above observations make CeCN_5_ as well as potentially TbCN_3_ and TbCN_5_ compounds of high interest for investigations of many-electron effects in these systems at the higher level of the electronic structure theory, e.g., within the DFT + DMFT.^53^ An accurate theoretical description of the electronic structure and vibrational and structural properties of the compounds with occupied *f*-states is therefore outside of the scope of the present study.

The volume-pressure dependences of the DFT-relaxed structures of LaCN_3_ and CeCN_5_ in the pressure range of 0–120 GPa and 10–120 GPa, respectively, were fitted with a third-order Birch–Murnaghan equation of state (Figure 4). The obtained bulk moduli (*K*_0_(LaCN_3_) = 192.5(6) GPa, *K*_0_(CeCN_5_) = 250.7(14) GPa) are higher than the bulk moduli of known nitrides and carbides of lanthanum (LaN,^58^ La_2_C_3_,^59^ LaC_2_^59^) and cerium (CeN,^60^ CeC,^59^ Ce_2_C_3_,^59^ CeC_2_^59^) due to the presence of robust polycarbonitride 3D frameworks with relatively incompressible short C–N and N–N bonds. At the same time, the obtained bulk moduli are lower than the bulk moduli of the ultraincompressible C–N solids,^17^ likely because of the dilution of the C–N framework with metal ions. The degree of this dilution is higher in the case of LaCN_3_ since the ratio between metal cations and the framework-forming C and N atoms is higher in LaCN_3_ than in CeCN_5_, which explains the significant difference in the bulk moduli of LaCN_3_ and CeCN_5_. Another reason for the enhancement of *K*_0_ in CeCN_5_ compared to the *K*_0_ of LaCN_3_ is the smaller size of the Ce^4+^ cation compared to that of La^3+^ and, as a consequence, shorter and less compressible metal-N bonds.

Considering the dynamical stability at 1 bar and the smooth behavior of the lattice parameters of DFT variable-cell relaxed structures down to ambient pressure, a first attempt to experimentally verify the recoverability of LaCN_3_ was undertaken. Upon sample decompression, a data point at 75(2) GPa was successfully collected (Table S8). The unit cell volume of LaCN_3_ at 75(2) GPa perfectly matches the DFT-calculated equation of state (Figure 4). However, when the pressure was decreased to 50(2) GPa, the sample chamber collapsed, and it was no longer possible to continue the experiment (see footnote of Table S8). After an unsuccessful decompression experiment, another DAC#4 was prepared and loaded with a lanthanum piece embedded in solid cyanuric triazide (C_3_N_12_). Cyanuric triazide serves as a precursor of nitrogen (and might be carbon as well) for the synthesis of LaCN_3_ as well as a “hard” solid pressure transmission medium. The same P and T conditions were used for the synthesis: the sample was compressed to 101(2) GPa and laser-heated to temperatures reaching up to 2500 K (Table S1). According to synchrotron single-crystal XRD, the LaCN_3_ phase was formed (Table S8). Subsequently, DAC#4 was decompressed, and LaCN_3_ was preserved in the cell down to 6(2) GPa. Notably, upon the decompression of DAC#4, the unit cell volume of LaCN_3_ lay systematically below the DFT-calculated equation of state curve, due to residual stresses in the hard pressure transmitting medium (Figure 4). Below 6(2) GPa, the cell was opened in an Ar glovebox and closed again to avoid an interaction with oxygen and moisture. According to the XRD measurements, LaCN_3_ was no longer present. Therefore, LaCN_3_ exists at least down to 6(2) GPa, but was not found recoverable under ambient conditions.

DAC#2, containing the CeCN_5_ compound, was first compressed from 90(2) to 115(2) GPa and then decompressed, allowing data collection from 115(2) to 33(2) GPa. Below that pressure, the sample escaped the experimental chamber, preventing further measurements from being done. In total, seven *P*–*V* data points were collected for the CeCN_5_ phase, found to persist even down to 33(2) GPa (Table S9). The experimental data agree well with the DFT-calculated equation of state (Figure 4). No decompression experiment was carried out for DAC#3, containing the TbCN_3_ and TbCN_5_ compounds.

**Figure 5:**
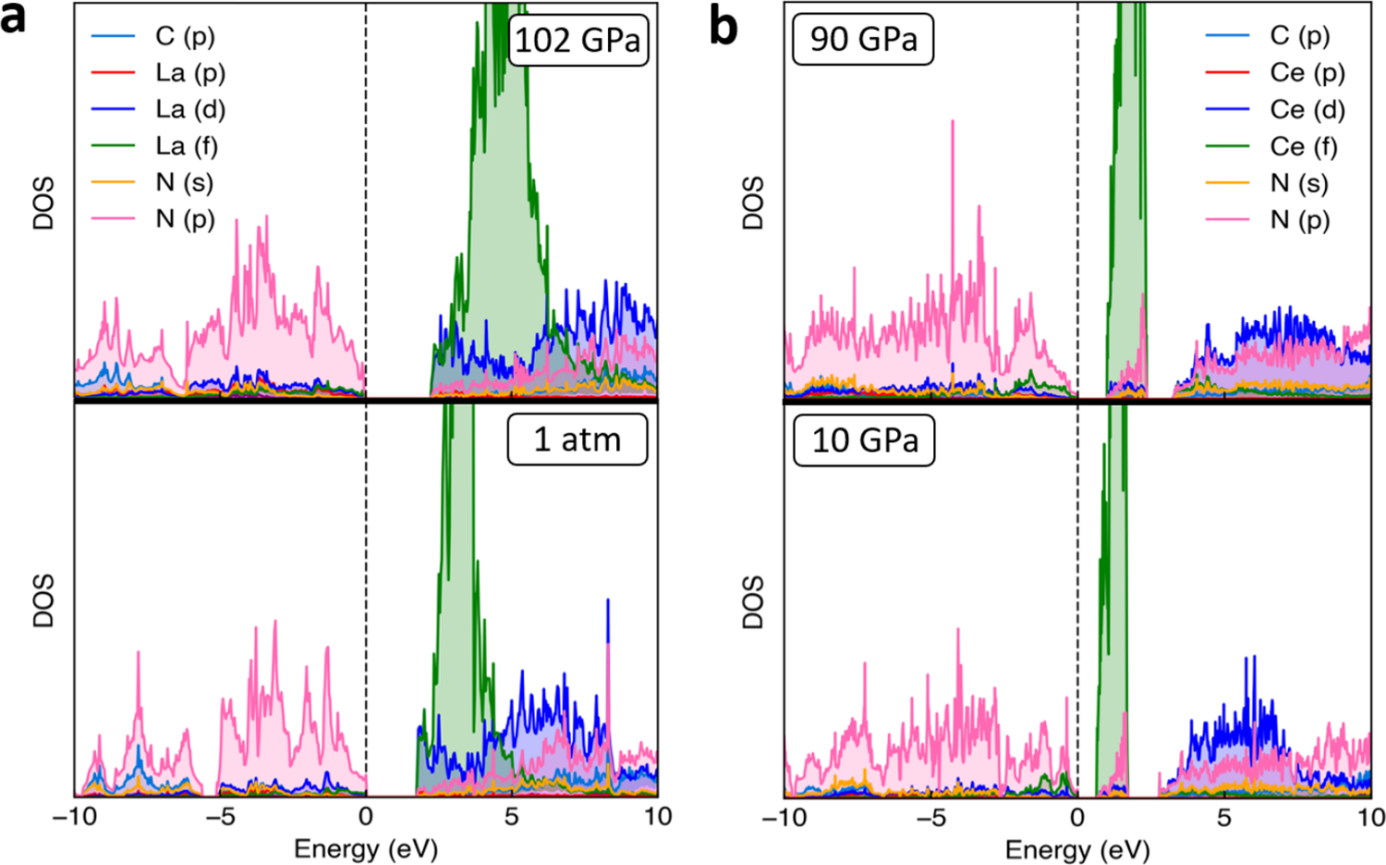
Electron density of states of (a) LaCN_3_ at 102 GPa and 1 bar, and (b) CeCN_5_ at 90 and 10 GPa.

The computed electron densities of states for LaCN_3_ and CeCN_5_ at the synthesis pressure and at low pressure are shown in Figure 5. LaCN_3_ has band gaps of 2.2 and 1.7 eV at 102 GPa and 1 atm, respectively. CeCN_5_ has a gap between the valence and conduction bands that is 3.2 eV at the synthesis pressure. Between the valence and conduction bands lie the unoccupied Ce 4f states. The gap from the valence band to the edge of the 4f band is 1 eV, and the 4f peak has a width of 1.4 eV. Upon decompression to 10 GPa, the band gap between the valence and conduction band decreases to 2.7 eV. The gap from the valence band to the 4f band edge is 0.6 eV, and the 4f peak has a width of 1.1 eV. The exact position of the unoccupied 4f peak is influenced by the choice of the *U* value. A higher value of *U* shifts the peak away from the Fermi energy. A larger value of *U* also slightly decreases the gap between the valence and conduction band. A similar effect of shifting the 4f peak and decreasing the band gap has also been observed in studies on CeO_2_.^51^ Both LaCN_3_ and CeCN_5_ compounds demonstrate a pressure-induced band gap opening.

## Conclusions

In this study, the four compounds LaCN_3_, TbCN_3_, CeCN_5_, and TbCN_5_ (first representatives of the class of polycarbonitrides) were synthesized in laser-heated diamond anvil cells at pressures between 90(2) and 111(2) GPa. Their crystal structures are built of a covalently single-bonded carbon–nitrogen anionic 3D framework consisting of CN_4_ tetrahedra connected via di- or oligo-nitrogen linkers. From a crystal chemistry perspective, the closest ambient condition analogues of these polycarbonitrides are Ln–Si–P metal silicon phosphide ternary compounds.

According to DFT calculations, the CeCN_5_ phase is dynamically stable down to 10 GPa and not stable under ambient conditions, while LaCN_3_ is (meta)stable under ambient conditions. While experiments did not validate the recoverability of the LaCN_3_ solid, one can expect that other high-pressure metal polycarbonitrides built of CN_4_ connected only by vertexes or partially N–N connected can be recoverable, analogously to the high-pressure C_3_N_4_ polymorphs, and CN_2_ compound containing N–N bonds. LaCN_3_ and CeCN_5_ are found to be semiconductors with a pressure-mediated band gap opening.

Due to the presence of hard polycarbonitride 3D frameworks with relatively incompressible short C–N and N–N bonds, LaCN_3_ and CeCN_5_ exhibit good mechanical properties with the bulk moduli higher than the bulk moduli of known nitrides or carbides of lanthanum and cerium. At the same time, the bulk moduli of lanthanide polycarbonitrides are lower than the bulk moduli of the ultraincompressible C–N solids,^17^ and dictated by the degree of dilution of the C–N framework with metal ions having longer and more compressible Ln–N contacts.

## Methods

### Sample Preparation

BX90-type DACs^61^ equipped with Boehler-Almax type diamonds^62^ of 120 μm (DAC#1, DAC#2, DAC#4) and 80 μm (DAC#3) culet diameter were used in these experiments. The sample chambers were formed by preindenting rhenium gaskets, initially 200 μm in thickness, down to 15–18 μm and laser-drilling a hole of 60 μm (DAC#1, DAC#2, DAC#4) or of 40 μm (DAC#3) in diameter in the center of the indentation. Pieces of lanthanide metals (La, Ce, Tb, 99.9%, Sigma-Aldrich) were placed in the sample chamber of individual DACs (DAC#1, DAC#2, DAC#3) directly in contact with one of the two diamond anvils, and molecular nitrogen was then loaded using high-pressure gas loading systems at the Bayerisch Geoinstitut (BGI, 1300 bar)^63^ or at the Centre for Science at Extreme Conditions (CSEC, 2000 bar). The Ce metal was loaded into the DAC in an Ar-filled glovebox, while La and Tb were loaded in air. DAC#4 was loaded with a lanthanum piece and solid cyanuric triazide (C_3_N_12_), and the lanthanum piece was placed in contact with one of the two diamond anvils. *Caution: Since the energetic cyanuric triazide compound is to some extent unstable against external stimuli, proper safety precautions should be taken especially when handling the materials in amounts exceeding those that are typically used for an experiment in a DAC. Laboratory personnel should wear protective equipment like grounded shoes, leather coats, Kevlar gloves, ear protection, and face shields*. The samples were compressed to their target pressure (Table S1) and laser-heated (λ = 1064 nm) up to 2500(500) K using a homemade laser-heating system at BGI^64^ or in CSEC. The temperatures reached during laser heating were determined by fitting the sample’s thermal emission spectrum to the gray body approximation of Planck’s radiation function in a given wavelength range (570–830 nm). The pressure in the DACs was determined using the Raman signal from the diamond anvils^65^ and additionally monitored by the X-ray diffraction signal of the Re gasket edge using rhenium’s equation of state.^66^

### X-ray Diffraction

The X-ray diffraction studies were conducted at the P02.2 beamline of Petra III, DESY (λ = 0.2904 Å); the ID11 beamline (λ = 0.2846 Å), the ID15b beamline (λ = 0.4099 Å), and the ID27 beamline (λ = 0.3738 Å) of the Extreme Brilliant Source European Synchrotron Radiation Facility (EBS-ESRF). At the P02.2 beamline of DESY, the X-ray beam was focused down to 2 × 2 μm^2^ and data was collected with a PerkinElmer 1621 XRD flat-panel detector. At the ID11 beamline of the ESRF, the X-ray beam was focused down to 0.75 × 0.75 μm^2^ and data was collected with an Eiger2X CdTe 4 M hybrid photon counting pixel detector. At the ID15b beamline of ESRF, the X-ray beam was focused down to 1.5 × 1.5 μm^2^ and data was collected with an Eiger2X CdTe 9 M hybrid photon counting pixel detector. At the ID27 beamline of the ESRF, the X-ray beam was focused down to 2 × 2 μm^2^ and data was collected with an Eiger2X CdTe 9 M hybrid photon counting pixel detector. In order to determine on which sample position the SCXRD data should be collected, a full X-ray diffraction mapping of the pressure chamber was performed. The sample position displaying the most and the strongest single-crystal reflections belonging to the phase of interest was chosen for the collection of single-crystal data, collected in step scans of 0.5° from −36 to +36°. The CrysAlis^Pro^ software package^67^ was used for the analysis of the SCXRD data (peak hunting, indexing, data integration, frame scaling, and absorption correction). To calibrate the instrument model in the CrysAlis^Pro^ software, i.e., the sample-to-detector distance, detector’s origin, offsets of the goniometer angles, and inclination of both the X-ray beam and detector with regard to the instrument axis, a single crystal of orthoenstatite [(Mg_1.93_Fe_0.06_)(Si_1.93_,Al_0.06_)O_6_, *Pbca* space group, *a* = 8.8117(2) Å, *b* = 5.18320(10) Å, and *c* = 18.2391(3) Å] was used. The DAFi program was used for the search of groups of reflections belonging to individual single-crystal domains.^68^ With the OLEX2 software package,^69^ structures were solved with the ShelXT structure solution program^70^ using intrinsic phasing and refined with the ShelXL^71^ refinement package with the least-squares minimization. Crystal structure visualization was made with the VESTA software.^72^

### Theoretical Calculations

Calculations were carried out by the Vienna Ab initio simulation package (VASP),^50^ using the projector augmented wave (PAW) method.^73^Table S10 contains information about the used POTCARs. For LaCN_3_, the GGA-PBE functional was used in standard DFT formalism.^74^ For CeCN_5_, due to the presence of localized 4f electrons, the L(S)DA + *U* formalism as formulated by Dudarev et al. was employed to account for the on-site Coulomb repulsion.^75^ The label (S) in L(S)DA + *U*, was omitted for simplicity. In the Dudarev et al. formalism, the LDA + *U* functional takes the form
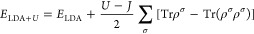
where ρ is the density matrix of 4f states, and *U* and *J* are the spherically averaged matrix elements of the screened Coulomb electron–electron interaction.^75^ Only the difference between *U* and *J* is significant, and thus, *U* – *J* is simply denoted as *U*. For CeCN_5_, a *U* = 6 eV was used. The value of *U* was chosen based on a semiempirical approach to agree with the experimental values of the lattice constants. The value 6 eV is also justified since values between 5 and 8 eV have been used in previous studies on, for example, pure Ce, Ce impurities in Ag and Pd, and CeO_2_.^51,54−57,76^ For both LaCN_3_ and CeCN_5_ compounds, spin-polarized calculations were employed to account for possible local magnetic moments. Calculations for structure relaxations and eDOS were done for the LaCN_3_ and CeCN_5_ compounds with a 6 × 6 × 6 *k*-point mesh according to the Monkhorst–Pack method.^77^ For the relaxations, a Gaussian smearing was used with a smearing of 0.03 eV. For eDOS calculations, the tetrahedron method of Blöchl was used.^78^ The convergence criteria for the energy were set to 10^–7^ eV, and in structure relaxations, the forces on each ion were less than 10^–3^ eV/Å. These settings were used for both LaCN_3_ and CeCN_5_. The plane-wave cutoffs were set to 680 eV for LaCN_3_ and 740 eV for CeCN_5_. Phonons were calculated with the finite displacement method, with displacements generated by phonopy.^79,80^ For both LaCN_3_ and CeCN_5_, 2 × 2 × 2 supercells were used with a k-mesh of 4 × 4 × 4 *k*-points. Displacement amplitudes were 0.01 Å, as a default phonopy setting.

## Supporting Information Available

Summary of the performed DAC laser heating experiments; crystallographic information; dependence of the unit cell parameters of LaCN_3_ and CeCN_5_ with pressure; phonons dispersions; and supplementary discussion of CeCN_5_ compound with occupied 4f states (PDF)
