# Supplementary material for: Synthesis of LaCN3, TbCN3, CeCN5, and TbCN5 Polycarbonitrides at Megabar Pressures
Source: J Am Chem Soc. 2024 Jun 25;146(26):18161–71. doi: 10.1021/jacs.4c06068 (PMC11229003; doi:10.1021/jacs.4c06068)
Supplement: Supplementary file 1 — ja4c06068_si_001.pdf [file ja4c06068_si_001.pdf]

Supporting information

Synthesis of $LaCN_3$, $TbCN_3$, $CeCN_5$, and $TbCN_5$ Polycarbonitrides at Megabar Pressures

Andrey Aslandukov[1,2*], Akun Liang[3*], Amanda Ehn[4], Florian Trybel[4], Yuqing Yin[4], Alena Aslandukova[1], Fariia I. Akbar[1], Umbertoluca Ranieri[3], James Spender[3], Ross T. Howie[3], Eleanor Lawrence Bright[5], Jonathan Wright[5], Michael Hanfland[5], Gaston Garbarino[5], Mohamed Mezouar[5], Timofey Fedotenko[6], Igor A. Abrikosov[4], Natalia Dubrovinskaia[2,4] Leonid Dubrovinsky[1], Dominique Laniel[3]

[1] Bavarian Research Institute of Experimental Geochemistry and Geophysics (BGI), University of Bayreuth, 95440 Bayreuth, Germany

[2] Material Physics and Technology at Extreme Conditions, Laboratory of Crystallography, University of Bayreuth, 95440 Bayreuth, Germany

[3] Centre for Science at Extreme Conditions and School of Physics and Astronomy, University of Edinburgh, EH9 3FD Edinburgh, United Kingdom

[4] Department of Physics, Chemistry and Biology (IFM), Linköping University, SE-581 83, Linköping, Sweden

[5] European Synchrotron Radiation Facility, 38000 Grenoble, France

[6] Photon Science, Deutsches Elektronen-Synchrotron, 22607 Hamburg, Germany

*Equal contribution. Correspondence to andrii.aslandukov@uni-bayreuth.de and aliang@exseed.ed.ac.uk

Supplementary Tables

Table S1. Summary of the performed DAC laser heating experiments and their reaction conditions for the synthesis of polycarbonitrides.

Sample	Reaction mixture	Temperature, K	Pressure, GPa	Polycarbonitride reaction product
DAC#1	La + N_2 + $C_{diamond\ anvil}$	2500(500)	102(2)	$LaCN_3$
DAC#2	Ce + N_2 + $C_{diamond\ anvil}$	2500(500)	90(2)	$CeCN_5$
DAC#3	Tb + N_2 + $C_{diamond\ anvil}$	2500(500)	111(2)	$TbCN_3$, $TbCN_5$
DAC#4	La + CTA[1] + $C_{diamond\ anvil}$	2500(500)	102(2)	$LaCN_3$

[1]CTA is cyanuric triazide (C_3N_{12})

Table S2. Structure refinement details of $LaCN_3$ at 102(2) GPa. The full crystallographic data was deposited to the ICSD under the deposition number CSD 2352230.

Chemical formula	$LaCN_3$
Temperature (K)	293
Pressure (GPa)	102(2)
**Crystal data**	
Mr	192.95
ρ (g/cm^3)	8.573
Crystal system, space group	orthorhombic, *Pnma*
a (Å)	4.1059(12)
b (Å)	4.870(5)
c (Å)	7.4758(15)
V (Å^3)	149.49(16)
Z	4
Radiation type	X-ray, λ = 0.2846 Å
μ (mm^{-1})	12.802
**Data collection**	
No. of measured, independent and observed [I > 2σ(I)] reflections	473/198/152
R_{int}	4.30%
$(\sin θ/λ)_{max}$ (Å^{-1})	0.769
**Refinement**	
R[$F^2 > 4σ(F^2)$], wR(F^2), GOF	3.67%, 8.31%, 1.078
data/parameters ratio	198/17
$Δρ_{max}$, $Δρ_{min}$ (e Å^{-3})	2.140, -2.327

Atomic positions and equivalent isotropic (or isotropic) ADPs

Atom	Wyckoff site	Fractional atomic coordinates			U_{eq} or U_{iso} (Å^2)
		x	y	z	
La1	4*c*	0.08304(18)	0.25	0.62043(11)	U_{eq} = 0.0057(4)
C1	4*c*	0.012(3)	0.25	0.1698(17)	U_{iso} = 0.003(2)
N1	8*d*	0.413(2)	0.007(4)	0.4178(9)	U_{iso} = 0.0042(13)
N2	4*c*	0.366(3)	0.25	0.1686(14)	U_{iso} = 0.004(2)

Table S3. Structure refinement details of $TbCN_3$ at 111(2) GPa. The full crystallographic data was deposited to the ICSD under the deposition number CSD 2352231.

Chemical formula			$TbCN_3$		
Temperature (K)			293		
Pressure (GPa)			111(2)		
**Crystal data**					
Mr			212.96		
ρ (g/cm^3)			10.380		
Crystal system, space group			orthorhombic, *Pnma*		
a (Å)			3.9813(15)		
b (Å)			4.7305(12)		
c (Å)			7.2358(15)		
V (Å^3)			136.28(7)		
Z			4		
Radiation type			X-ray, λ = 0.4099 Å		
μ (mm^{-1})			11.798		
**Data collection**					
No. of measured, independent and observed [I > 2σ(I)] reflections			254/144/114		
R_{int}			3.49%		
$(\sin θ/λ)_{max}$ (Å^{-1})			0.853		
**Refinement**					
R[$F^2 > 4σ(F^2)$], wR(F^2), GOF			4.09%, 9.94%, 1.103		
data/parameters ratio			144/17		
$Δρ_{max}$, $Δρ_{min}$ (e Å^{-3})			1.729, -2.407		
**Atomic positions and equivalent isotropic (or isotropic) ADPs**					
Atom	Wyckoff site	Fractional atomic coordinates			U_{eq} or U_{iso} (Å^2)
		x	y	z	
Tb1	4*c*	0.0863(4)	0.25	0.61802(12)	U_{eq} = 0.0091(4)
C1	4*c*	0.025(7)	0.25	0.172(3)	U_{iso} = 0.009(4)
N1	8*d*	0.413(6)	0.501(2)	0.4159(16)	U_{iso} = 0.006(2)
N2	4*c*	0.379(6)	0.25	0.155(3)	U_{iso} = 0.009(3)

Table S4. Structure refinement details of $CeCN_5$ at 90(2) GPa. The full crystallographic data was deposited to the ICSD under the deposition number CSD 2352232.

Chemical formula				$CeCN_5$	
Temperature (K)				293	
Pressure (GPa)				90(2)	
**Crystal data**					
Mr				222.18	
ρ (g/cm^3)				7.657	
Crystal system, space group				monoclinic, $P2_1/n$	
a (Å)				3.8889(9)	
b (Å)				4.7394(7)	
c (Å)				10.487(6)	
β (°)				94.41(4)	
V (Å^3)				192.72(13)	
Z				4	
Radiation type				X-ray, λ = 0.28457 Å	
μ (mm^{-1})				10.553	
**Data collection**					
No. of measured, independent and observed [I > 2σ(I)] reflections				723/423/304	
R_{int}				4.21%	
$(\sin θ/λ)_{max}$ (Å^{-1})				0.833	
**Refinement**					
R[F^2 > 4σ(F^2)], wR(F^2), GOF				3.26%, 6.26%, 0.913	
data/parameters ratio				423/34	
$Δρ_{max}$, $Δρ_{min}$ (e Å^{-3})				1.851, -1.270	
**Atomic positions and equivalent isotropic (or isotropic) ADPs**					
Atom	Wyckoff site	Fractional atomic coordinates			U_{eq} or U_{iso} (Å^2)
		x	y	z	
Ce1	4*e*	0.1469(2)	0.8419(1)	0.1545(1)	U_{eq} = 0.0074(2)
C1	4*e*	0.2350(30)	0.1574(17)	0.6261(15)	U_{iso} = 0.0083(12)
N1	4*e*	0.1470(30)	0.9171(14)	0.7001(15)	U_{iso} = 0.0092(14)
N2	4*e*	0.0340(20)	0.1486(15)	0.5112(12)	U_{iso} = 0.0071(11)
N3	4*e*	0.0890(20)	0.3548(15)	0.1255(11)	U_{iso} = 0.0077(11)
N4	4*e*	0.1410(30)	0.4090(13)	0.6833(15)	U_{iso} = 0.0081(13)
N5	4*e*	0.2280(30)	0.2175(14)	0.0207(14)	U_{iso} = 0.0105(14)

Table S5. Structure refinement details of $TbCN_5$ at 111(2) GPa. The full crystallographic data was deposited to the ICSD under the deposition number CSD 2352233.

Chemical formula					TbCN₅
Temperature (K)					293
Pressure (GPa)					111(2)
**Crystal data**					
Mr					240.98
ρ (g/cm^3)					8.950
Crystal system, space group					monoclinic, $P2_1/n$
a (Å)					3.8334(2)
b (Å)					4.5221(9)
c (Å)					10.3516(5)
β (°)					94.699(5)
V (Å^3)					178.84(4)
Z					4
Radiation type					X-ray, λ = 0.4099 Å
μ (mm^{-1})					9.022
**Data collection**					
No. of measured, independent and observed [I > 2σ(I)] reflections					428/286/235
R_{int}					2.92%
(sin θ/λ)$_{max}$ (Å^{-1})					0.866
**Refinement**					
R[F^2 > 4σ(F^2)], wR(F^2), GOF					4.48%, 11.32%, 1.004
data/parameters ratio					286/34
$\Delta\rho_{max}$, $\Delta\rho_{min}$ (e Å^{-3})					2.498, -2.322
**Atomic positions and equivalent isotropic (or isotropic) ADPs**					
Atom	Wyckoff site	Fractional atomic coordinates			U_{eq} or U_{iso} (Å^2)
		x	y	z	
Tb1	4*e*	0.15275(12)	0.8298(3)	0.15535(5)	U_{eq} = 0.0047(5)
C1	4*e*	0.238(3)	0.180(5)	0.6250(12)	U_{iso} = 0.007(2)
N1	4*e*	0.147(2)	0.926(4)	0.7006(9)	U_{iso} = 0.0040(17)
N2	4*e*	0.041(3)	0.155(4)	0.5068(12)	U_{iso} = 0.009(2)
N3	4*e*	0.090(3)	0.338(4)	0.1240(10)	U_{iso} = 0.0042(19)
N4	4*e*	0.138(3)	0.427(5)	0.6887(11)	U_{iso} = 0.009(2)
N5	4*e*	0.219(3)	0.208(5)	0.0188(10)	U_{iso} = 0.0072(19)

Table S6. Experimentally determined crystallographic data for $LaCN_3$ at 102(2) GPa in comparison with the corresponding DFT-relaxed structure. Note that pressure was fixed to 102 GPa in the theoretical simulations, while the volume of the unit cell, lattice parameters, and equilibrium state parameters were calculated.

	Exp.			Calc.		
**Space group**	*Pnma*			*Pnma*		
**Volume**	149.49(16) Å^3			148.63 Å^3		
**Lattice parameters**	a = 4.1059(12) Å<br>b = 4.870(5) Å<br>c = 7.4758(15) Å			a = 4.0900 Å<br>b = 4.8537 Å<br>c = 7.4873 Å		
**Atomic positions**	La1	x	0.08304(18)	La1	x	0.0815
		y	0.25		y	0.25
		z	0.62043(11)		z	0.6219
	C1	x	0.012(3)	C1	x	0.0210
		y	0.25		y	0.25
		z	0.1698(17)		z	0.1690
	N1	x	0.413(2)	N1	x	0.4165
		y	0.007(4)		y	0.0084
		z	0.4178(9)		z	0.4190
	N2	x	0.366(3)	N2	x	0.3689
		y	0.25		y	0.25
		z	0.1686(14)		z	0.1664

Table S7. Experimentally determined crystallographic data for $CeCN_5$ at 90(2) GPa in comparison with the corresponding DFT-relaxed structure. Note that pressure was fixed to 90 GPa in the theoretical simulations, while the volume of the unit cell, lattice parameters and equilibrium state parameters were calculated.

	**Exp.**			**Calc.**		
**Space group**	$P2_1/n$			$P2_1/n$		
**Volume**	192.72(13) Å^3			191.66 Å^3		
**Lattice parameters**	a = 3.8889(9) Å<br>b = 4.7394(7) Å<br>c = 10.487(6) Å<br>β = 94.41(4)°			a = 3.8874 Å<br>b = 4.7240 Å<br>c = 10.471 Å<br>β = 94.59°		
**Atomic positions**	Ce1	x	0.1469(2)	Ce1	x	0.1502
		y	0.8419(1)		y	0.8422
		z	0.1545(1)		z	0.1540
	C1	x	0.2350(30)	C1	x	0.2600
		y	0.1574(17)		y	0.1553
		z	0.6261(15)		z	0.6300
	N1	x	0.1470(30)	N1	x	0.1534
		y	0.9171(14)		y	0.9191
		z	0.7001(15)		z	0.7002
	N2	x	0.0340(20)	N2	x	0.0343
		y	0.1486(15)		y	0.1484
		z	0.5112(12)		z	0.5119
	N3	x	0.0890(20)	N3	x	0.0928
		y	0.3548(15)		y	0.3554
		z	0.1255(11)		z	0.1250
	N4	x	0.1410(30)	N4	x	0.1435
		y	0.4090(13)		y	0.4063
		z	0.6833(15)		z	0.6867
	N5	x	0.2280(30)	N5	x	0.2295
		y	0.2175(14)		y	0.2252
		z	0.0207(14)		z	0.0231

Table S8. The lattice parameters of $LaCN_3$ at different pressures.

DAC	Pressure, GPa	a, Å	b, Å	c, Å	Volume, Å^3
DAC#1	102(2)[1]	4.1059(12)	4.870(5)	7.4758(15)	149.49(16)
DAC#1	75(2)[2]	4.170(3)	4.950(4)	7.592(10)	156.7(3)
DAC#4	101(2)[1]	4.1252(10)	4.8659(13)	7.448(2)	149.49(7)
DAC#4	85(2)	4.111(8)	4.906(5)	7.523(6)	151.7(3)
DAC#4	64(2)	4.202(5)	4.905(4)	7.632(5)	157.3(2)
DAC#4	37(2)	4.233(5)	5.109(6)	7.652(13)	165.5(4)
DAC#4	23(2)	4.335(3)	5.141(2)	7.851(2)	174.98(16)
DAC#4	6(2)	4.4406(5)	5.3417(7)	8.1055(16)	192.27(5)

[1]Pressure of synthesis.

[2]After the compound's synthesis at 102(2) GPa, the DAC was decompressed from that pressure down to 75(2) GPa. At the next pressure point, 50(2) GPa, the nitrogen pressure transmitting medium went out through microcracks in the diamond resulting in a diamond breaking and the complete gasket closure, with the pressure dropping to 1 atm. The sample could not be found afterwards.

Table S9. The lattice parameters of $CeCN_5$ at different pressures.

Pressure, GPa	a, Å	b, Å	c, Å	β, °	Volume, Å^3
90(2)[1]	3.889(1)	4.739(1)	10.487(6)	94.41(4)	192.7 (2)
114(2)[2]	3.812(1)	4.645(2)	10.464(6)	93.95(3)	184.9(2)
95(2)	3.857(7)	4.707(4)	10.490(8)	93.56(6)	189.4(2)
69(2)	3.9250(16)	4.7804(16)	10.611(5)	94.25(4)	198.5(2)
58(2)	3.9514(10)	4.8448(12)	10.710(3)	94.80(2)	204.3(1)
49(2)	3.9583(12)	4.8827(13)	10.831(6)	94.54(3)	208.7(2)
33(2)	4.0081(15)	4.935(5)	10.960(2)	94.51(2)	216.1(3)

[1]Pressure of synthesis.

[2]After synthesis at 90(2) GPa, the DAC was compressed first to 114(2) GPa, and then decompressed from that pressure down to 33(2) GPa. Below that pressure, one of the diamonds broke, and the sample escaped.

Table S10. POTCARs used for $LaCN_3$ and $CeCN_5$.

Compound	Functional	Element	Core	Number of valence electrons
$LaCN_3$	PBE	La	[Kr4d]	11
		C	s2p2	4
		N	s2p3	5
$CeCN_5$	LDA	Ce	[Kr4d]	12
		C	s2p2	4
		N	s2p3	5

Supplementary Figures

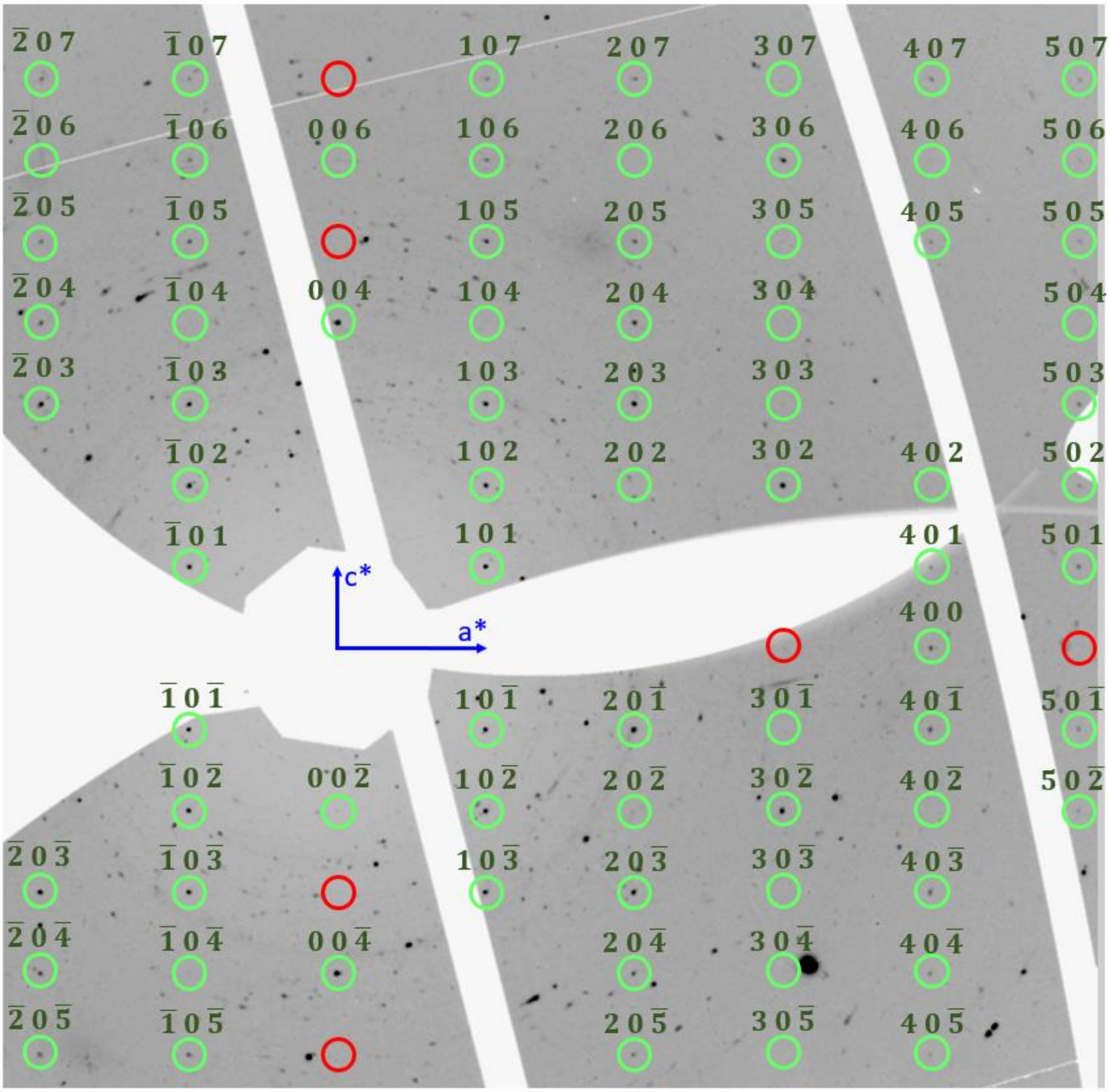

Figure S1. (*h 0 l*) reciprocal lattice plane of $LaCN_3$ at 102(2) GPa reconstructed from the experimental SCXRD dataset in the CrysAlis^{Pro} software. The reflections circled in green correspond to the crystallite of $LaCN_3$ whose structure was determined. The red circles illustrate the positions of the *h* 0 0 reflections with *h* = 2n + 1 and the 0 0 *l* reflections with *l* = 2n + 1, which are systematically absent in the *Pnma* space group. The reflections that are not encircled belong to other crystallites of the same $LaCN_3$ phase or of other binary La-N phases present in the multiphase multigrain sample.

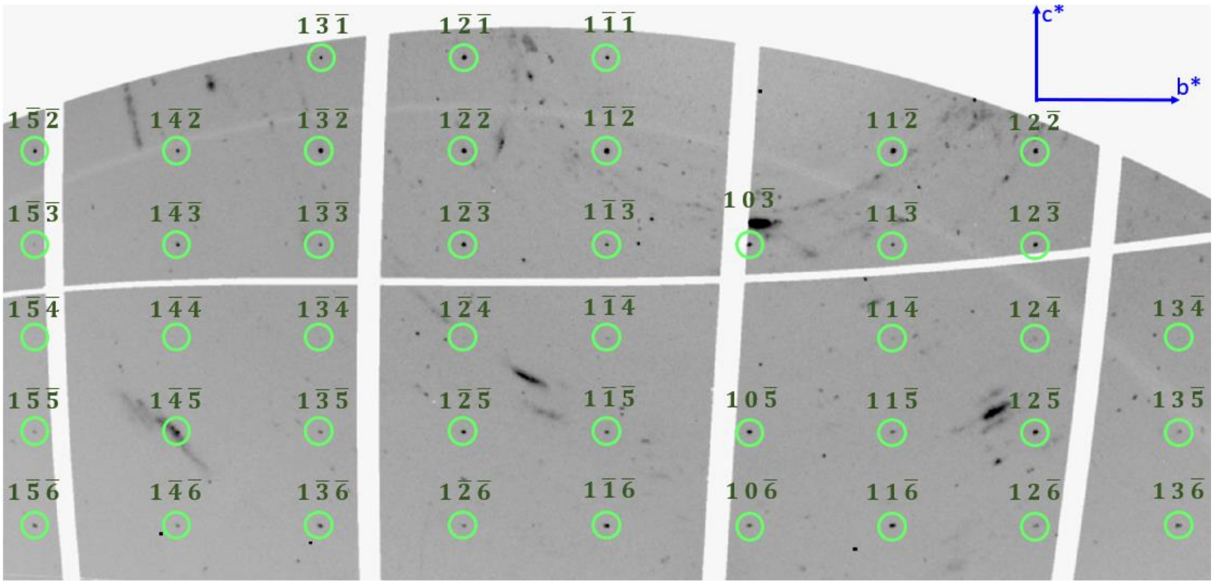

Figure S2. (1 *k l*) reciprocal lattice plane of $TbCN_3$ at 111(2) GPa reconstructed from the experimental SCXRD dataset in the CrysAlis^{Pro} software. The reflections circled in green correspond to the crystallite of $TbCN_3$ whose structure was determined. The reflections that are not encircled belong to other crystallites of the same $TbCN_3$ phase or of other binary Tb-N phases and $TbCN_5$ present in the multiphase multigrain sample.

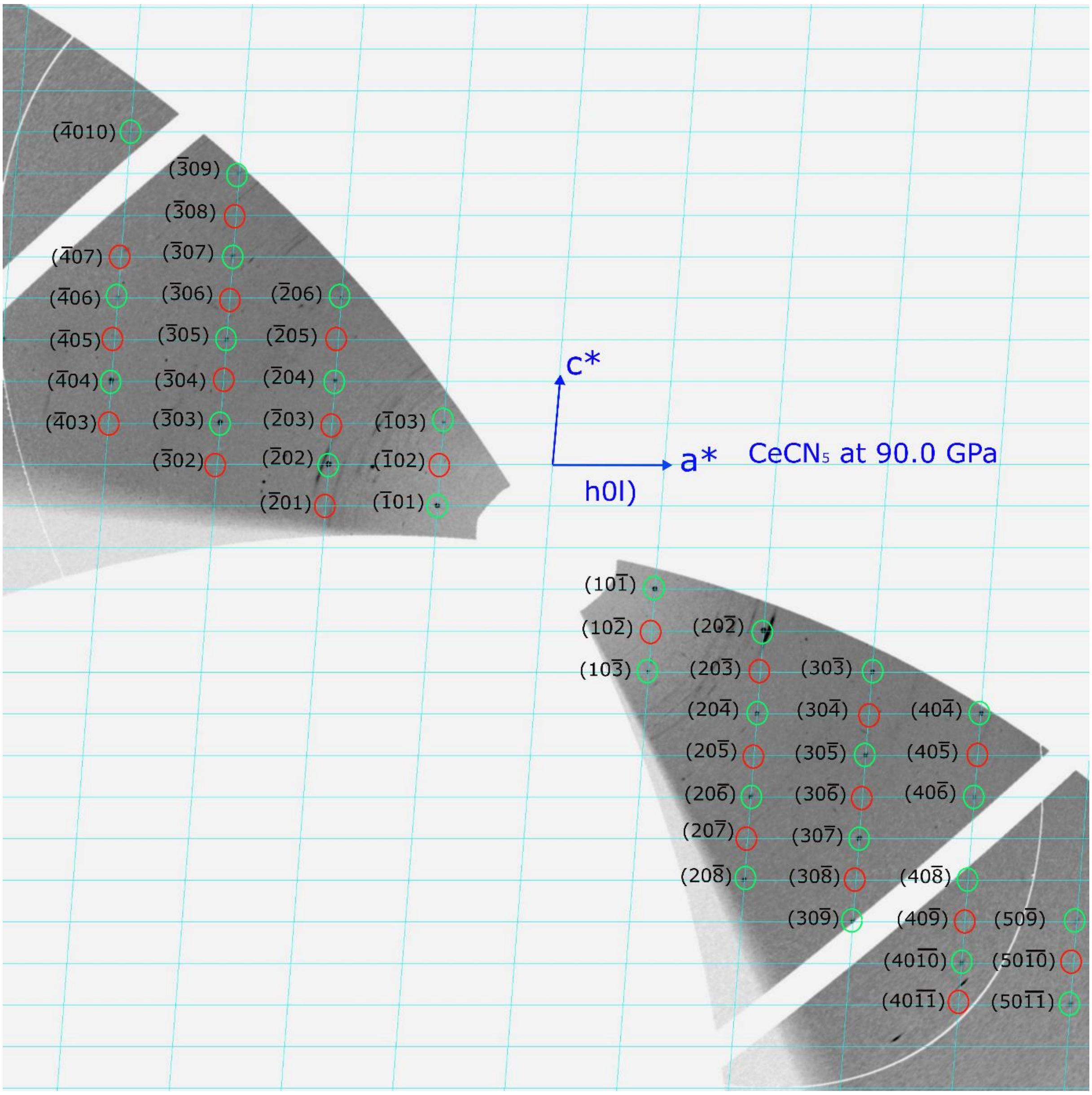

Figure S3. (*h 0 l*) reciprocal lattice plane of $CeCN_5$ at 90(2) GPa reconstructed from the experimental SCXRD dataset in CrysAlis^{Pro} software. The reflections circled in green correspond to the crystallite of $CeCN_5$ whose structure was determined. The red circles illustrate the positions of the *h* 0 *l* reflections with $h+l$ = 2n + 1, which are systematically absent in the $P2_1/n$ space group. The reflections that are not encircled belong to other crystallites of the same $CeCN_5$ phase or of other binary Ce-N phases present in the multiphase multigrain sample.

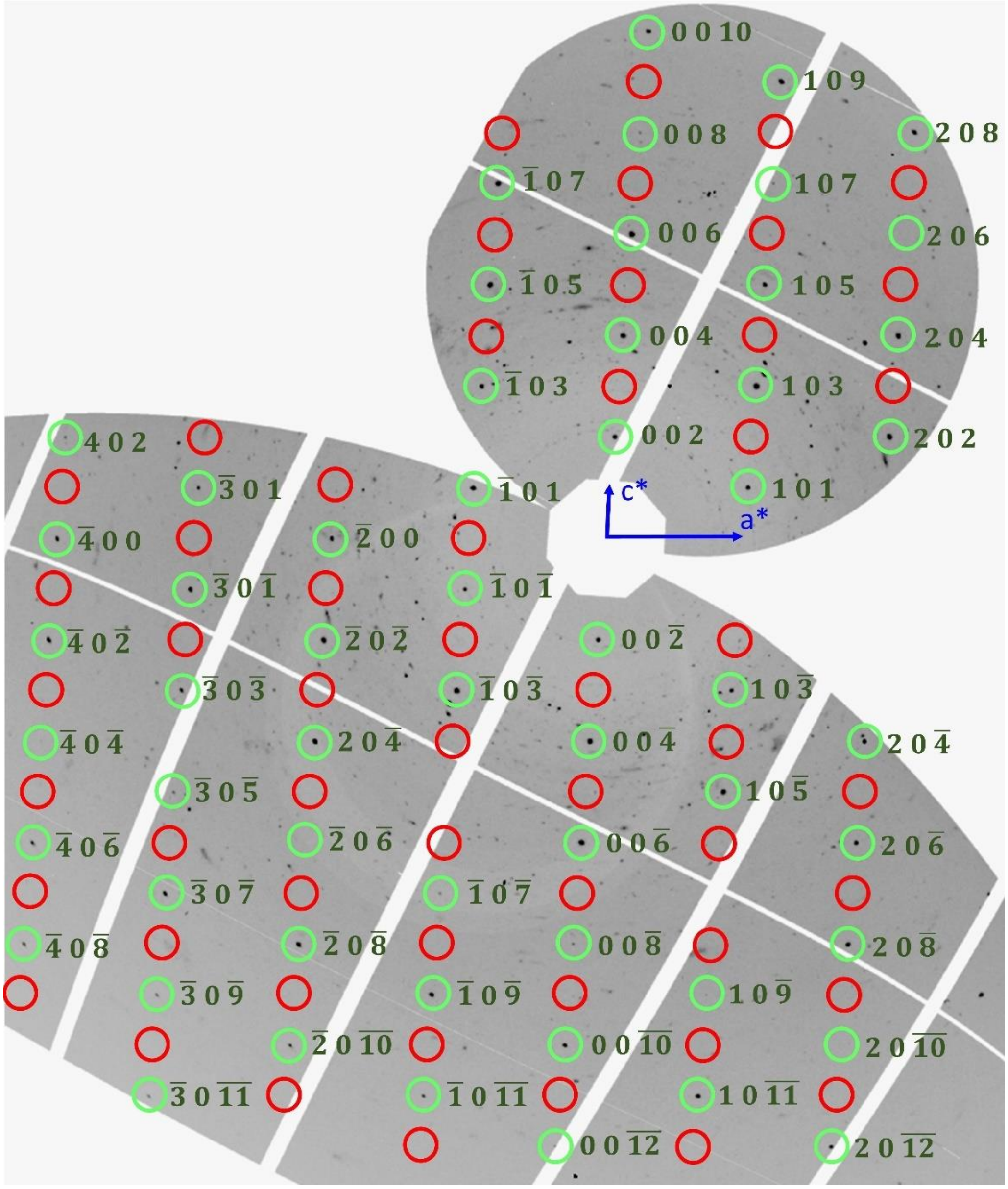

Figure S4. (*h 0 l*) reciprocal lattice plane of $TbCN_5$ at 90(2) GPa reconstructed from the experimental SCXRD dataset in CrysAlis^{Pro} software. The reflections circled in green correspond to the crystallite of $TbCN_5$ whose structure was determined. The red circles illustrate the positions of the *h* 0 *l* reflections with $h+l = 2n + 1$, which are systematically absent in the $P2_1/n$ space group. The reflections that are not encircled belong to other crystallites of the same $TbCN_5$ phase or of other binary Tb-N phases and $TbCN_3$ present in the multiphase multigrain sample.

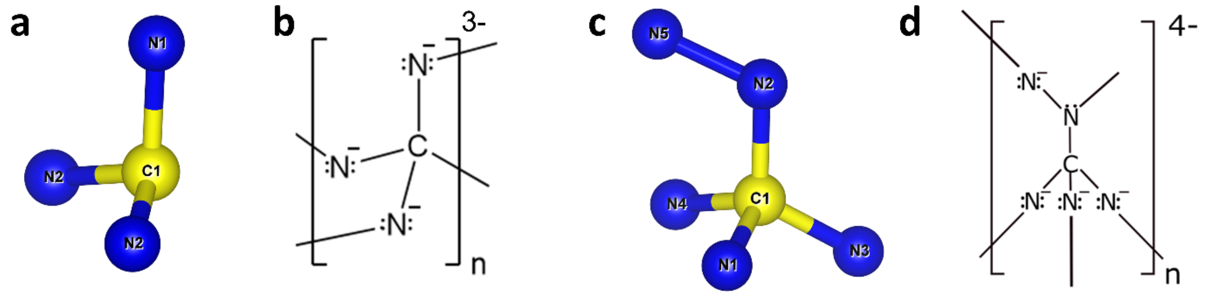

Figure S5. (a) A representation of the repeating CN_3 unit in $LaCN_3$/$TbCN_3$. (b) Lewis formula of the $[CN_3]^{3-}_{\infty}$ anion in $LaCN_3$/$TbCN_3$. (c) A representation of the repeating CN_5 unit in $CeCN_5$/$TbCN_5$. (d) Lewis formula of the $[CN_5]^{4-}_{\infty}$ anion in $CeCN_5$/$TbCN_5$.

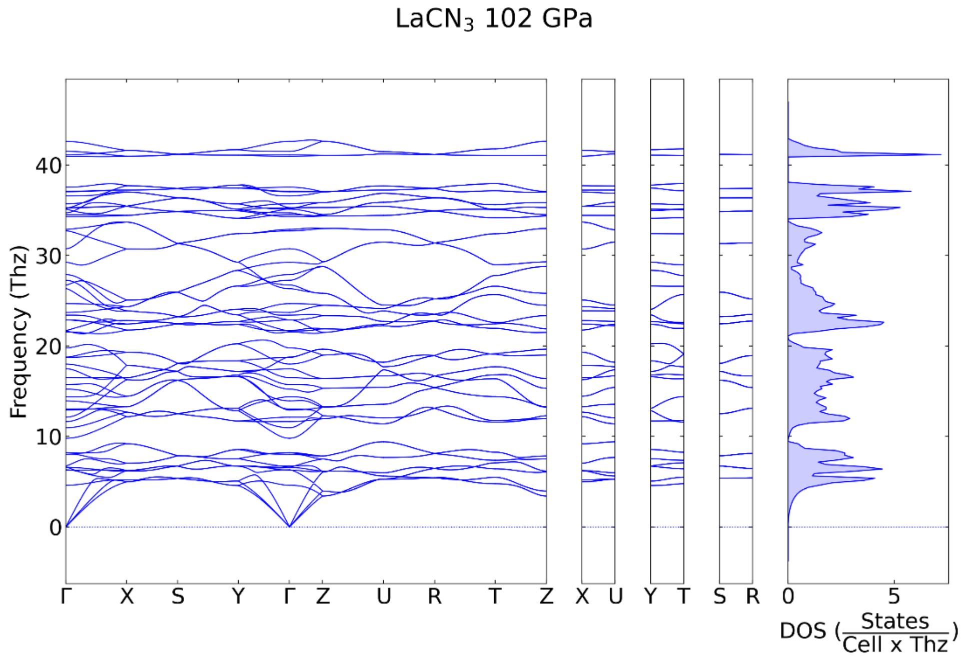

(a)

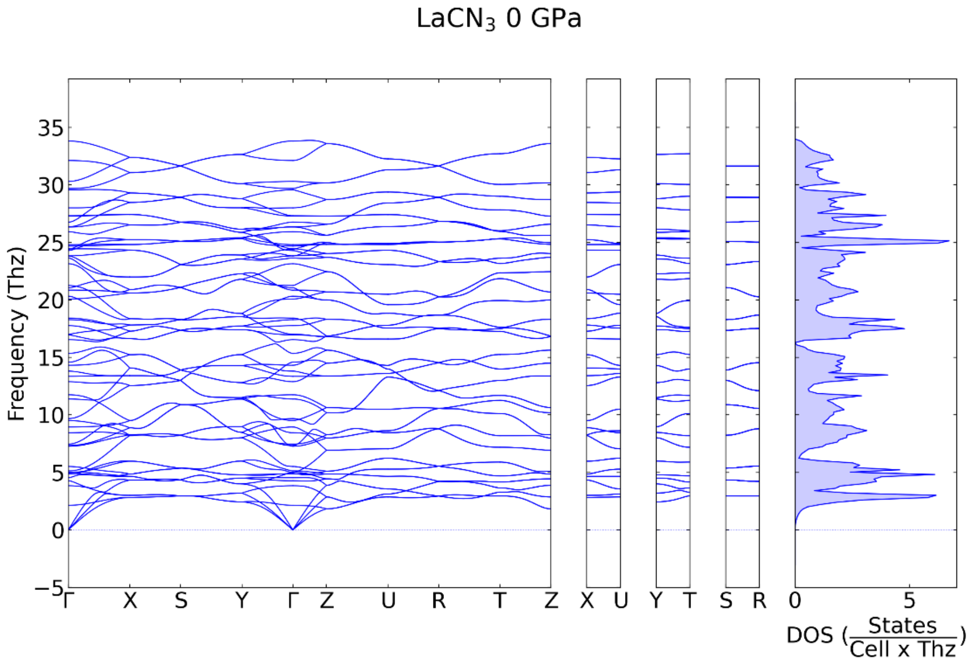

(b)

Figure S6. Calculated phonon dispersions of $LaCN_3$ at (a) 102 GPa and (b) 1 atm.

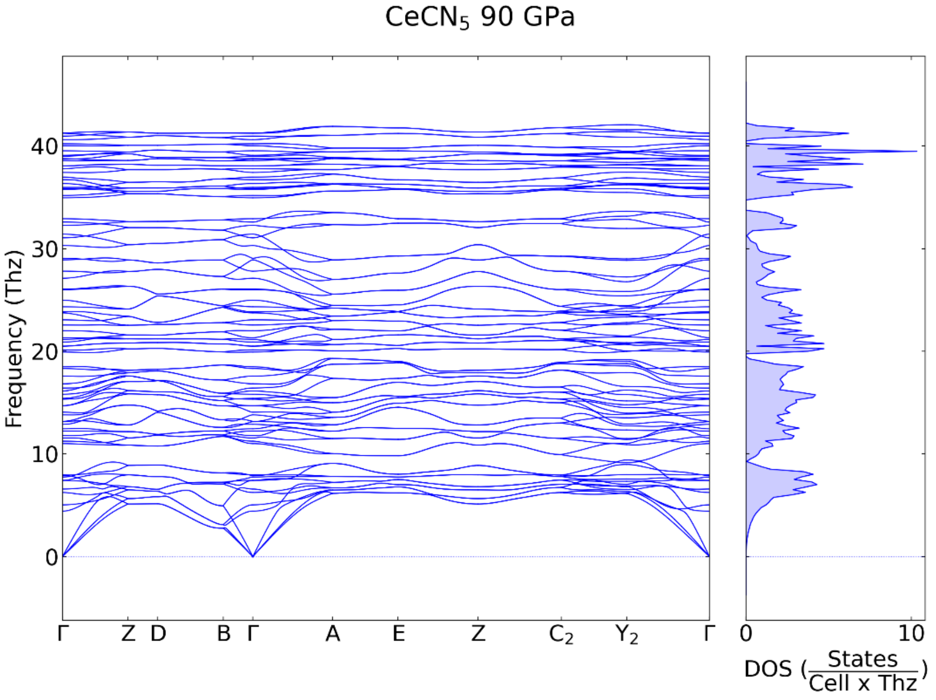

(a)

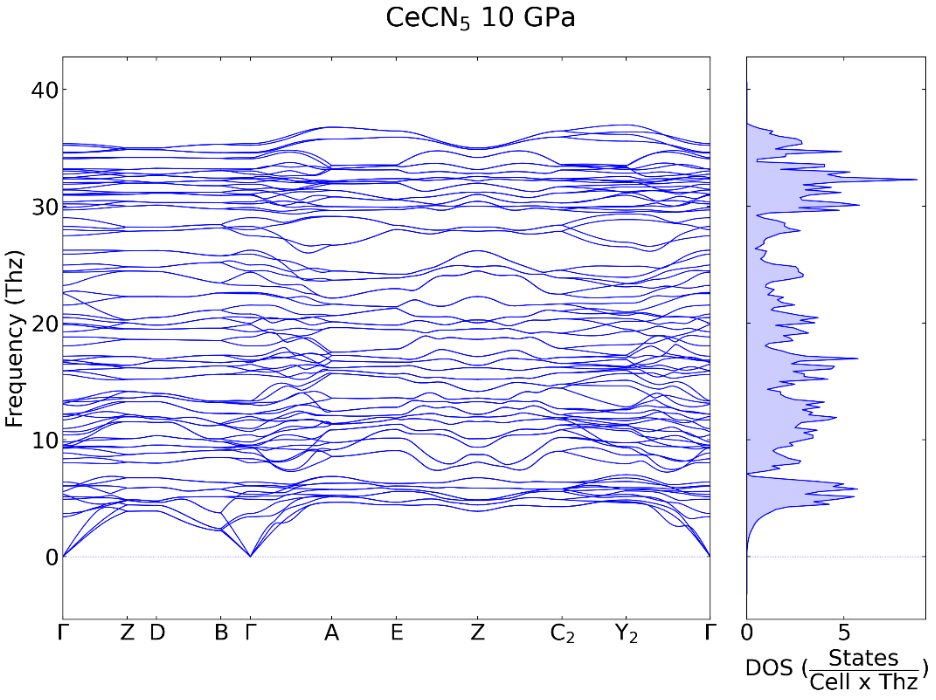

(b)

Figure S7. Calculated phonon dispersions of $CeCN_5$ at (a) 90 GPa and (b) 10 GPa.

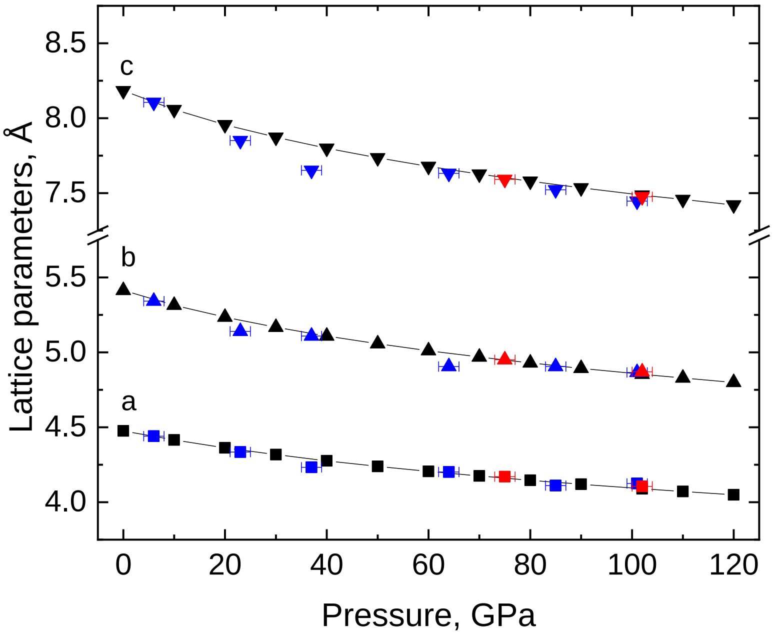

Figure S8. The dependence of the unit cell parameters of $LaCN_3$ with pressure. The black symbols represent calculated data points obtained from DFT while the red and blue symbols represent experimental data points obtained from SCXRD data collected in DAC#1 and DAC#4, respectively.

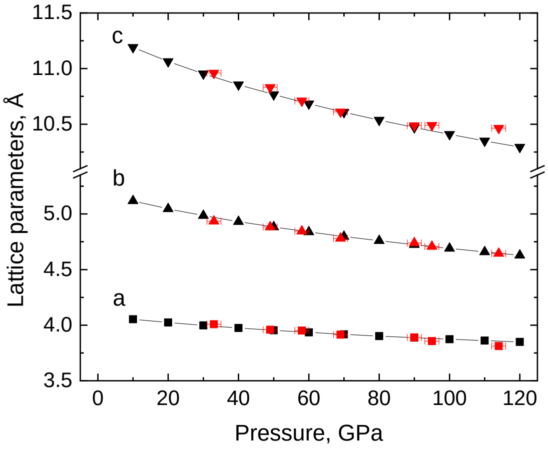

(a)

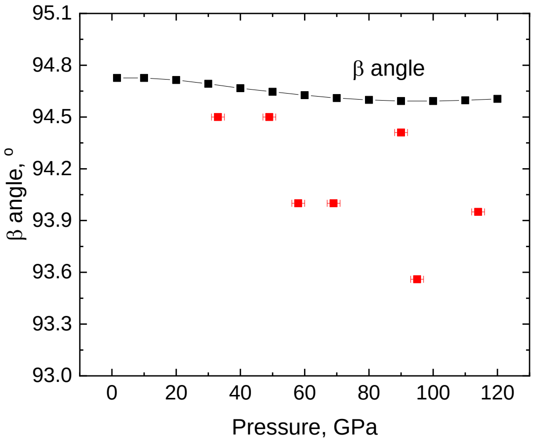

(b)

Figure S9. The dependence of the unit cell parameters of $CeCN_5$ with pressure: (a) *a*, *b*, *c* lattice parameters and (b) *β* angle. The black symbols represent calculated data points obtained from DFT, the red symbols represent experimental data points obtained from SCXRD data.

Supplementary discussion of $CeCN_5$ compound with occupied *4f*-states.

$CeCN_5$ is a *4f* electron system, with pure Ce having one *4f* electron. The cerium oxidation state in the $CeCN_5$ compound is expected to be +4 (see main paper). The presence of *4f* electrons requires careful consideration of the many-electron effects, especially when the states are occupied. Previous studies on the rare-earth elements, to which Ce belongs, suggest that local density approximation (LDA) and semi-local generalized gradient approximation (GGA) within DFT may not be sufficient to treat these materials. In this study, the LDA+U method was employed (see Methods, Theoretical Calculations).

At a pressure of 10 GPa and above, all *4f* states of $CeCN_5$ are unoccupied according to our calculations. Using LDA+U for $CeCN_5$, insulating state was obtained in the pressure range from synthesis pressure down to 10 GPa (**Fig. 5b** in the main text). Comparison of structural parameters for $CeCN_5$ shows that the computed results are in very good agreement with experimental data (**Table S7**). This indicates that LDA+U is likely sufficient to describe the structural properties of this material in the high-pressure phase.

At pressures below 10 GPa, LDA+U simulations result in an electronic transition, upon which one of the *f* electron state becomes occupied (**Fig. SD1**), and $CeCN_5$ becomes metallic. The material also gains a magnetic moment, specifically originating from the one *4f* electron. In this level of theory, the transition is accompanied by a rapid change of structural parameters (**Figs. SD2 and SD3**). For an accurate theoretical study of this transition, a higher-level theory is required, such as DFT+DMFT,[1] which can treat many-electron effects in strongly correlated systems, such as $CeCN_5$ with partly occupied *f*-states.

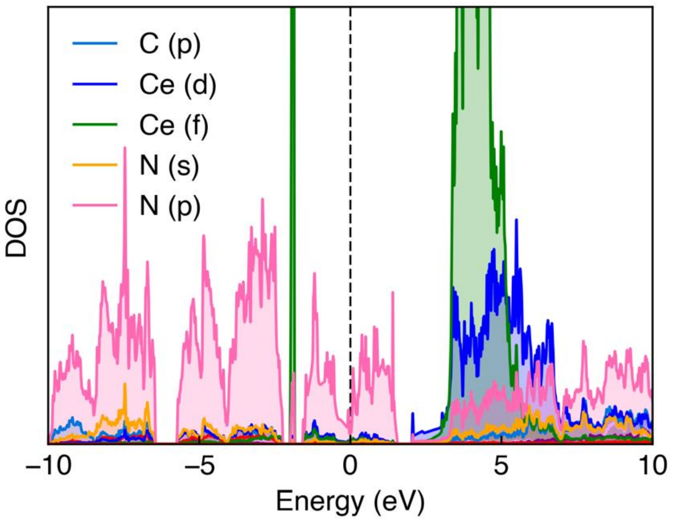

Figure SD1. The electron density of states of $CeCN_5$ at 1 atm.

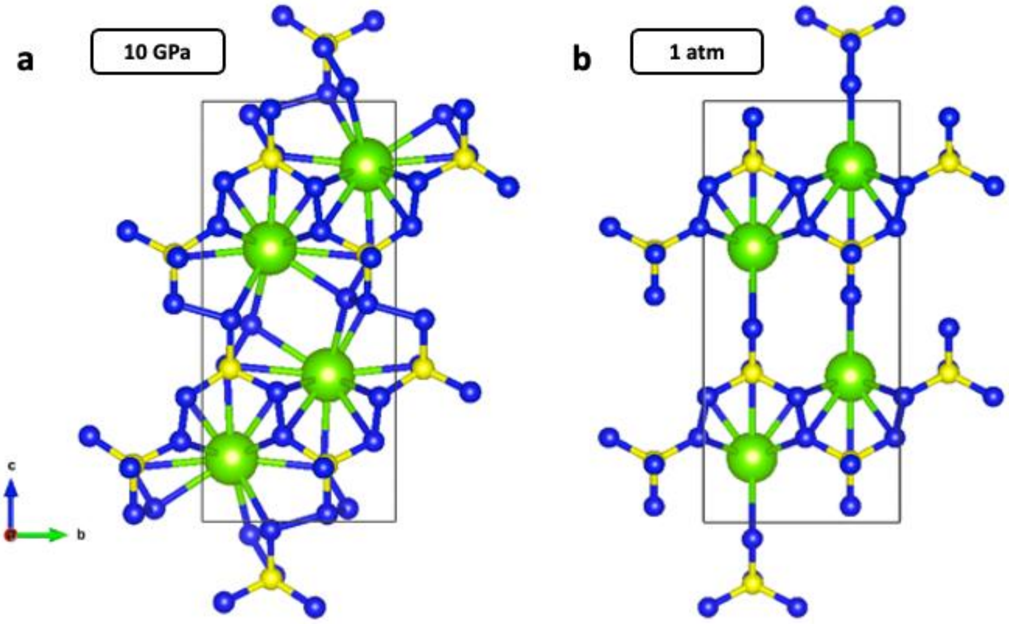

Figure SD2. Crystal structure of $CeCN_5$ at 10 GPa and at 1 atm, with an electronic and structural transition having occurred in the latter.

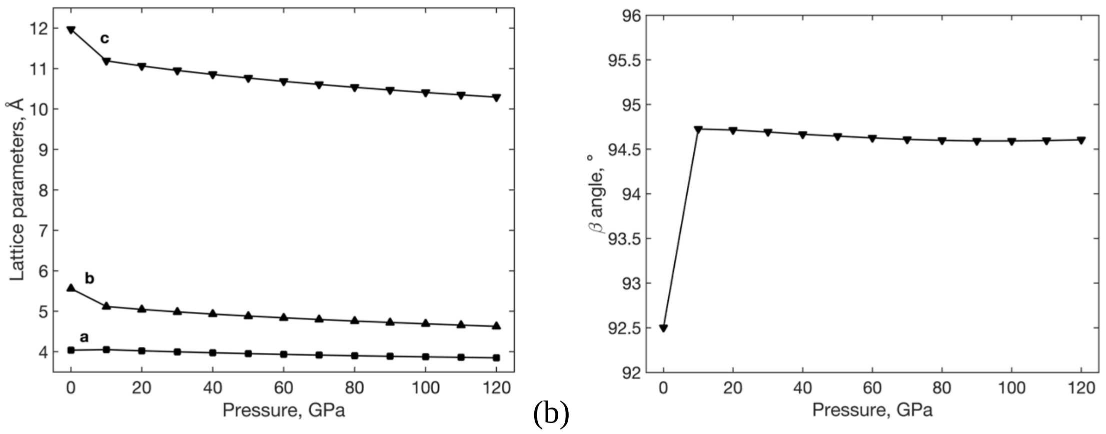

(a) (b)

Figure SD3. The dependence of the unit cell parameters of $CeCN_5$ with pressure: (a) lattice parameters a, b, and c and (b) β-angle.

References

1. Georges, A., Kotliar, G., Krauth, W. & Rozenberg, M. J. Dynamical mean-field theory of strongly correlated fermion systems and the limit of infinite dimensions. *Rev. Mod. Phys.* **68**, 13–125 (1996).
